# Machine Learning Models for Predicting Polymer Solubility in Solvents across Concentrations and Temperatures

**DOI:** 10.1021/acs.jpcb.4c06500

**Published:** 2024-12-12

**Authors:** Mona Amrihesari, Joseph Kern, Hilary Present, Sofia Moreno Briceno, Rampi Ramprasad, Blair Brettmann

**Affiliations:** † School of Chemical and Biomolecular Engineering, Georgia Institute of Technology, Atlanta, Georgia 30332, United States; ‡ School of Materials Science and Engineering, Georgia Institute of Technology, Atlanta, Georgia 30332, United States; § School of Computational Science and Engineering, Georgia Institute of Technology, Atlanta, Georgia 30332, United States

## Abstract

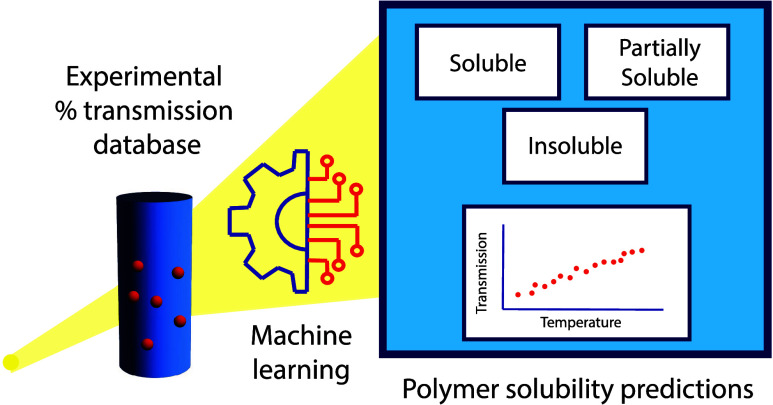

Artificial intelligence and machine learning have become essential tools in predicting material properties to aid in the accelerated design of new materials. Polymer solubility, critical for new formulations and solution processing, is one such property. However, current models are limited by inadequate experimental data sets that cannot capture the complexity and detail for many features contributing to polymer solubility. Here, we provide a data set for polymer solution behavior based on Crystal16 turbidity measurements that includes high quality percent transmission data for polymer solutions for a variety of polymers, solvents, concentrations and temperatures. We use this data set to train a model that predicts the experimental transmission data at many temperatures and multiple concentrations. From this, we are able to classify the polymer/solvent pairs into three solubility categories providing a level of granularity to predictions beyond prior binary classification models considering only solvent/nonsolvent classes. The inclusion of multiple concentrations, temperatures and partially soluble data expands solubility prediction capability beyond prior work into predictions more attractive for use by formulators and process designers working with industrial polymer solutions.

## 1 Introduction

In recent decades, the rapid advancement of artificial intelligence (AI) and machine learning (ML) has prompted significant interest in using ML methodologies to design materials meeting property and performance requirements across several application domains.^1^ Polymers are particularly interesting in materials science due to their important industrial applications and inherently complex characteristics^2,3^ and they have widespread application in sectors including single use plastics,^4^ functional coatings,^5^ pharmaceuticals,^6^ textiles,^7,8^ and more. Although they impart desirable properties to materials, they present complex attributes such as broad molecular weight distributions,^9^ large temperature-dependent morphological changes,^10^ and a semicrystalline nature with a processing-dependent degree of crystallinity. These attributes of polymers create challenges for developing high quality data sets to use with AI and ML and for accurately predicting material properties. We recently showed that information-rich data related to polymer solubility can be collected with a high degree of control using a parallel crystallizer with turbidity measurements.^11^ Here, we leverage that capability to create a large data set of percent transmission for polymer–solvent pairs as a function of concentration and temperature. We then assess the ability of ML models trained on this data to predict the percent transmission and translate those predictions into classifications of “soluble”, “insoluble”, and “partially soluble”.

Homogeneous polymer solutions are integral to a wide array of industrial processes, spanning membrane production,^12^ paint and coating formulation,^13^ and pharmaceutical development.^14^ The dissolution of polymers in solvents is a complex process influenced by factors including the thermodynamic driving forces, often characterized in part through the χ interaction parameter^15−18^ and kinetic factors, including phenomena such as surface erosion versus swelling dissolution mechanisms and solvent diffusion rates.^19,20^ While the “like dissolves like” rule-of-thumb provides a foundational principle for solubility prediction, it often overlooks critical factors such as temperature, molecular weight, and concentration, which directly impact solubility phenomena. The Working Party of Thermodynamics and Transport Properties of the European Federation of Chemical Engineering recently noted that there was a lack of “high-quality data in the literature for the solubility of larger molecules in solvents”, highlighting the challenge of solving industrially relevant problems related to solubility.^21^

Given the importance of polymer phase behavior and the breadth of factors that impact the observed solubility, there is a significant effort to develop new methods for solubility prediction. These include predicting the classification of solubility (solvent vs nonsolvent for a polymer–solvent pair),^22−24^ phase diagrams,^25^ thermodynamic parameters including Hansen solubility parameters,^26^ the χ interaction parameter and activity coefficients,^17^ and specific values of polymer solubility.^27^ The progress and emerging methods in this area were recently reviewed in Ethier et al.^28^ These predictions include purely data-driven, computational, and combination approaches, but most are limited by an insufficient amount of high-quality data, the same problem noted by the Working Party of Thermodynamics and Transport Properties of the European Federation of Chemical Engineering.^21^ This challenge motivates us to develop a well-controlled data set with polymer phase behavior information, in this case captured by transmission versus temperature curves.

High-quality data can aid in reducing bias and enhancing the accuracy of a ML model’s outcome. However, the lack of a globally accepted approach for measuring the solubility of a polymer makes it impossible to compare the results of different methods in the same way. Different measurement techniques, including visual inspection,^29,30^ light scattering,^31−33^ and turbidity measurements,^34,35^ have been used to examine the phase behavior and solubility of polymers. Turbidity measurements are a promising method, as they are relatively simple to perform and can be integrated into automated measurement systems. Our prior work has shown that a standardized turbidity method can be used to collect information-rich data that, for systems with fast kinetics, leads to high throughput data collection.^11^ In turbidity analysis a laser passes through a sample of a standard size at a specific temperature and the percent transmission is measured. For polymer solutions, 100% transmission corresponds to a 1-phase solution and 0% transmission corresponds to a fully precipitated 2-phase solution, with partially soluble systems showing values in between. Our prior work used a Crystal16 parallel crystallizer, where, per manufacturer’s recommendations 85% and above transmission corresponds to fully soluble, and 10% and below corresponds to fully insoluble.^11^ With transmission versus temperature data, polymer/solvent pairs can be classified as “soluble” or “insoluble” in the solvent. Furthermore, the data can be collected for multiple concentrations and translated into phase diagrams. Studies have also used turbidity titration data to determine the solubility parameter of polymers.^36,37^ Thus, polymer solution turbidity data in the form of transmission vs time and temperature is promising as a starting point for building different prediction tools for polymer solution phase behavior with different types of output.

We present here a study that first describes the polymer solution transmission percent data set and analyzes the ability of a ML regression model to extrapolate the transmission versus temperature results to concentrations not present in the training data set. While the experiments are designed such that the system does not reach equilibrium at each temperature, the prediction of the transmission data enables practitioners to interpret the data that is most relevant to the experimental solubility applications of interest. Key challenges in using the experimental data resulting from turbidity measurements in ML models are discussed in detail. We then use the turbidity data to classify polymer/solvent pairs at specific concentrations into three bins: “soluble”, “insoluble”, and “partially soluble”. This improves on prior binary classification work that is limited to “solvent” or “nonsolvent”,^24^ in providing a third class that is not fully insoluble or soluble at that concentration at the given temperature. We are motivated by USP29-NF24^38^ from the pharmaceutical industry, which classifies active pharmaceutical ingredient solubility in 7 classes based on the parts solvent needed to dissolve one part solute: very soluble, freely soluble, soluble, sparingly soluble, slightly soluble, very slightly soluble and practically insoluble. More classification categories allow improved granularity for predictions that can be used by researchers selecting solvents. Overall, we provide a well-controlled experimental data set for transmission versus temperature of polymer solutions, which correlates to polymer solubility and demonstrates the performance of ML models in directly predicting the data and in correctly classifying the polymer/solvent pairs. This work can help address the data sparsity and low-quality data problems for polymer solubility, within the limits discussed in the analysis.

## 2 Data Set and Methods

### 2.1 Generation of the Experimental Data for the Data Set

**Figure 1:**
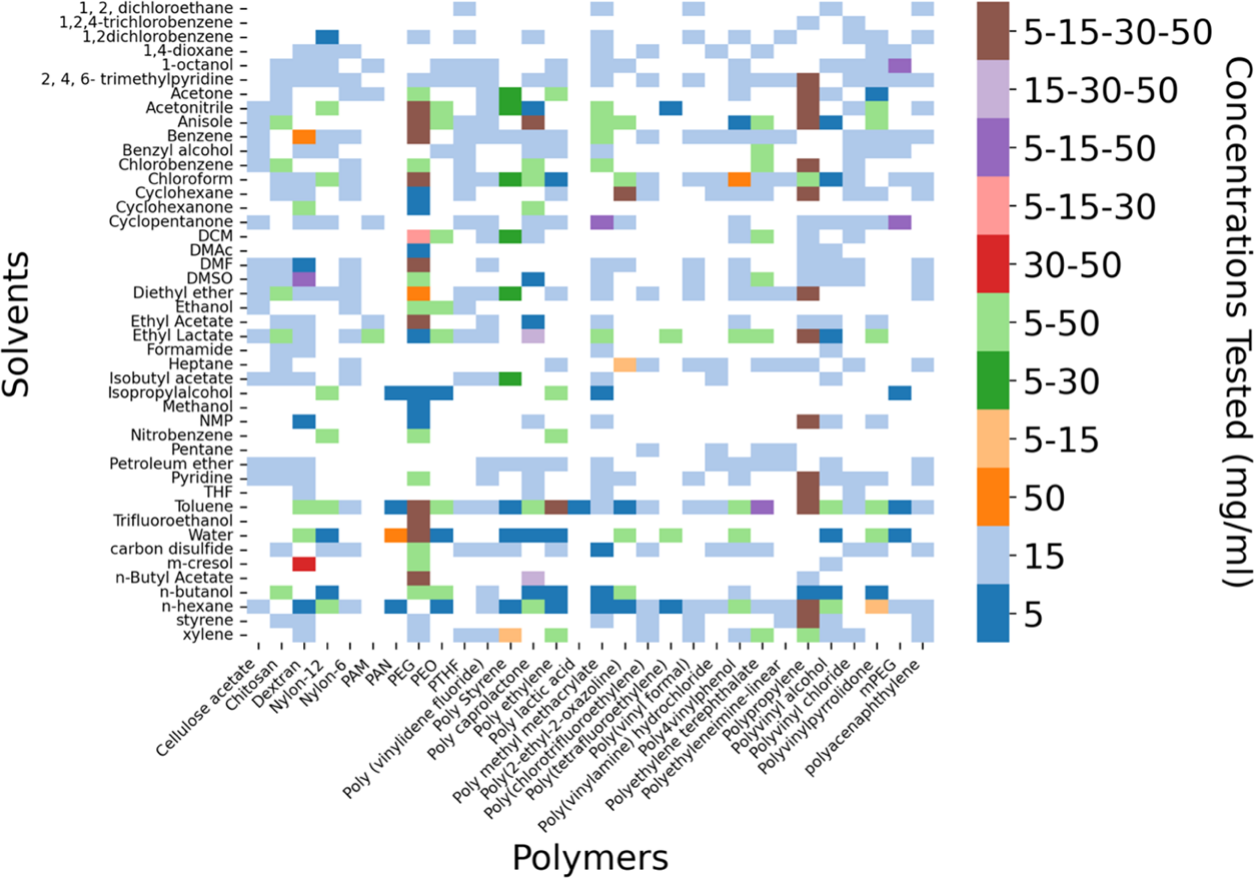
Processed data set used for modeling. The color of each data cell signifies the concentration tested. Different colors correspond to different concentrations tested for each polymer/solvent case in our data set. For instance, dark blue is linked to cases where only 5 mg/mL has been tested and brown is linked with cases where four concentrations of 5, 15, 30, and 50 mg/mL have been tested.

Solubility data for 30 polymers in 45 solvents was collected using a Crystal16 parallel crystallizer (Technobis Crystallization Systems), employing a 645 nm wavelength laser to measure turbidity. The Crystal16 equipment can concurrently process 16 samples in as many reactors. Figure 1 displays a heatmap of the collected data that was used for this manuscript, showcasing tests at concentrations of 5, 15, 30, and 50 mg/mL. These values were selected based on their relevance in solution processing and other polymer solution applications, though future expansion of the data set to broader concentration ranges would enable the model to be used for more scenarios. Most polymer–solvent combinations include at least a 15 mg/mL concentration. Poly(ethylene glycol) (PEG) and polypropylene (PP) have the most extensive data across all concentrations. 5.47% (27 tested) of polymer/solvent pairs were tested at 4 concentrations, 1.62% (8 tested) at 3 concentrations, 16.22% (80 tested) at 2 concentrations and 76.67% (378 tested) at 1 concentration. This is represented graphically in Figure 1. Occasionally other concentrations were tested and included in the data set, but they were not used in our experimental design so are not shown in Figure 1.

The polymers were chosen for diverse functional groups, with 74% having molecular weights below 15 000 Da and all having molecular weights above 5000 Da except for polyethylenimine-linear and polytetrahydrofuran (PTHF) having molecular weight of 2100 and 2900 Da, respectively. We selected a moderate molecular weight for testing, as it is well within the polymer regime but low enough to decrease kinetic effects and make it more likely the values measured match equilibrium values. Future studies could also include molecular weight as a parameter and the reported molecular weights for each polymer are included in the raw data, so could be included in analysis in the future. The polymers were mixed with the solvent either as purchased or after milling (milling performed for nylon-6, nylon-12, poly(ethylene terephthalate) (PET), polypropylene (PP), and polycaprolactone (PCL)). Solvents were also selected to have diverse properties, coming from the nonpolar (26%), polar protic (36%), and polar aprotic (38%) classes. Further information on polymers and solvents is listed in Supporting Tables S1 and S2 respectively.

**Figure 2:**
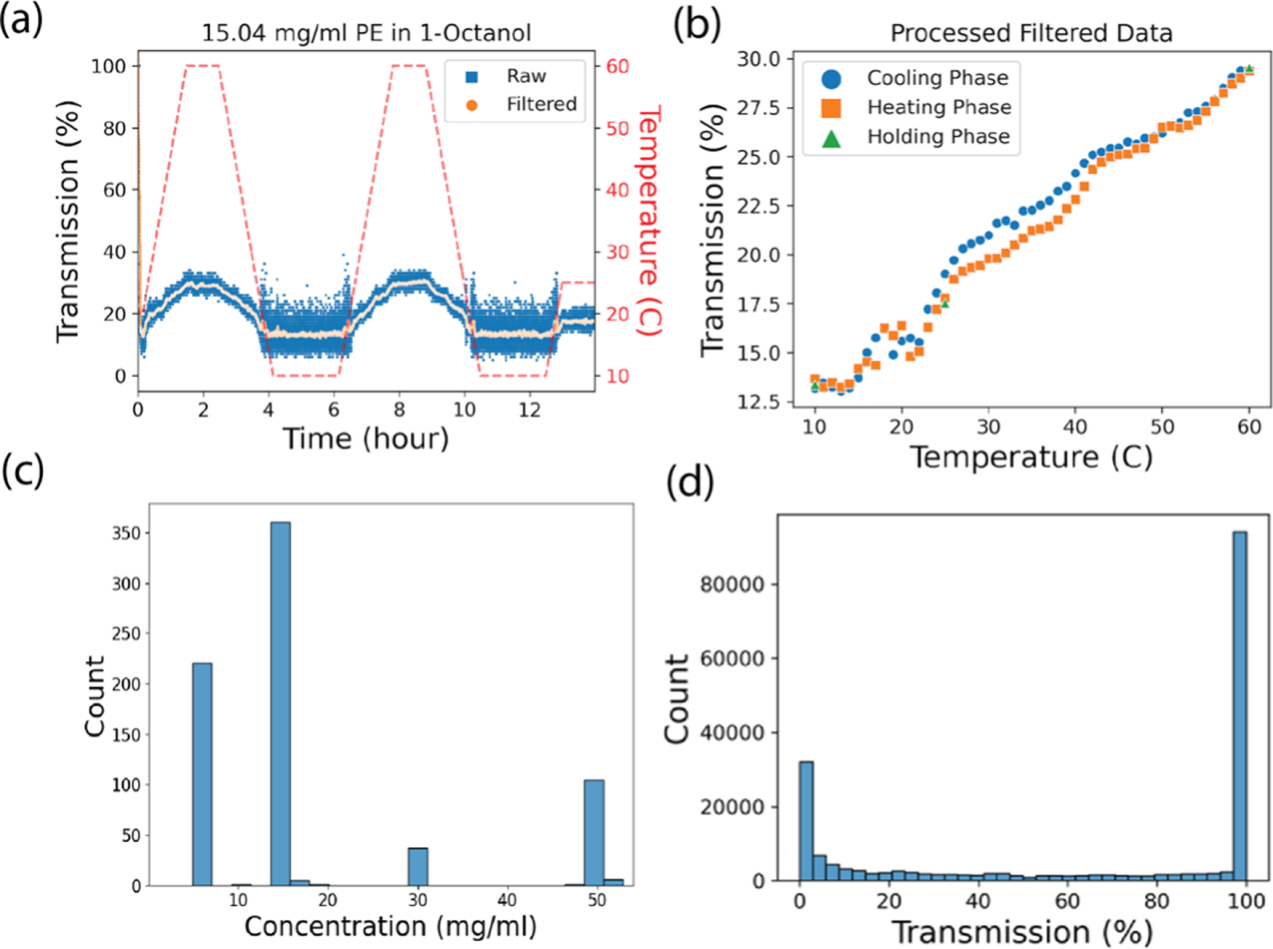
(a) Example of raw data collected from the Crystal16 instrument. The dashed-red line represents the temperature as a function of experiment time and the blue squares represent the transmission as a function of time. The orange circles represent the data after running the raw data through a Savitzky–Golay filter with a polynomial order of one. (b) Processed, filtered data from (a). The reported transmission vs temperature was found by averaging the transmission percentage in the heating, holding, or cooling phase, within integer steps of temperature ±0.5. (c) Distribution of concentrations tested among all postprocessed data. (d) Distribution of transmission among all postprocessed data.

The experiments involved two cycles of heating and cooling in 16 reactors, with hold times at cold (10 °C), hot (60 °C), and room temperatures (25 °C), as is as shown by the red dashed line in Figure 2a. The temperature ranged from 10 to 60 °C with a heating/cooling ramp rate of 0.5 °C/min, including hold times at 10 °C for 120 min, and at 60 and 25 °C for 60 min, which was found to be a sufficiently long to reach equilibrium for most polymer/solvent pairs.^11^ Although the data is not always at equilibrium, the ability to predict the turbidity data is a strong starting point for further analysis. It is important, however, to consider these predictions as nonequilibrium with the ramp rates of 0.5 °C/min. Our previous publication contains more thorough information on standardizing our approach to collecting the turbidity data.^11^

### 2.2 Data Curation

The Crystal16 method provides transmission versus temperature and time, as depicted in Figure 2a. Despite a discernible underlying trend in transmission values, certain samples exhibit intrinsic noise. In the sample shown in Figure 2a with the blue curve, this is particularly evident during the holding period, where the temperature remains constant. To mitigate this noise, we applied a Savitzky–Golay filter to the transmission data using the SciPy Python package (Figure 2a orange curve).^39^ We opted for a polynomial order of one, assuming a linear relationship between temperature and transmission. The window size for filtering encompassed 2 min of data, determined by averaging the time differences between consecutive timesteps in an experiment and rounding to the nearest integer representing 2 min of steps. To ensure an odd window size and balanced filtering around each data point, we adjusted the window size by adding one if the calculated value was even. This approach guarantees equal distance from the center on both sides of the filter.

After applying the filter, our aim was to consolidate the data into a unified transmission-versus-temperature plot, as depicted in Figure 2b. This involved discretizing the temperature into integer steps of ±0.5 °C and averaging the corresponding transmission values. However, achieving low uncertainty in averaged transmission values at specific temperatures is not always possible, especially during temperature ramps where kinetic effects can be slower than the ramping speed. Consequently, significant variations can arise in transmission-temperature profiles depending on whether the sample is undergoing a heating, cooling, or holding phase. To address this, we categorized temperature states accordingly and averaged values within each phase of the temperature profile. This variability in transmission-temperature relationships is evident between temperatures 22–40 °C in Figure 2b. While the disparity appears small in this sample, it can be substantial in others, reaching up to 100% transmission difference, as demonstrated between temperatures 32 and 45 °C in Figure S1(b), though these extremes are rare in the data set.

For our modeling purposes, we focused solely on the transmission values during the cooling phase. This decision stemmed from the observation of a considerable error during the initial temperature ramp-up cycle for a subset of reactors, as evidenced in Figure S1(a,b). This discrepancy was likely attributed to insufficient equilibration of these specific samples. Furthermore, out of the 923 reactors we analyzed, 129 exhibited significant noise during the holding periods of the experiment, as depicted in Figure S1(c,d). This observation was unexpected as these periods were anticipated to have a small standard deviation due to their extended duration. We hypothesized that this noise might be a result of experimental aberrations or exceptionally slow kinetics and consequently chose to exclude these periods from our analysis for this work.

For the remaining data set, we filtered out values exhibiting standard deviations exceeding 5% in the averaged transmission values. Most of these variations stemmed from fluctuations in the transmission versus temperature across different cooling cycles, as illustrated in Figure S1(b). Notably, these shifts were comparatively minor compared to the shifts observed between heating and cooling cycles. Out of the initial pool of 880 reactors subjected to our filtration and processing procedure, 138 were eliminated due to large standard deviations (>8% transmission) observed during the holding phase, while an additional four were excluded because all data points exhibited standard deviations exceeding five. This left us with 738 reactors for further analysis. Notably, the behavior of the four excluded reactors mirrored that of those removed due to high standard deviations in the holding phase, suggesting either exceptionally slow kinetics or abnormal experimental conditions.

Subsequently, we isolated the transmission versus temperature curves during the cooling phase of the ramping cycles. The resulting concentration and transmission distributions are depicted in Figure 2c,d and represent 39 407 data points comprised of 30 polymer samples and 45 unique solvents. Twenty-eight unique polymers were tested, and PEG was tested at two different molecular weights of 8 and 1000 kg/mol. To assess whether there is bias in which concentrations were removed we analyzed the decrease in data points from the Crystal16 unfiltered data to the final processed data set and found no strong bias for which concentrations were removed (see analysis in Supporting Information Section C).

### 2.3 Fingerprinting, Machine Learning Model Selection Methodology, and Hyperparameter Tuning

Our objective was to model transmission percentage as a function of temperature for a diverse spectrum of polymers, solvents, and concentrations. To numerically represent the 30 polymers in our study, we used a labeling scheme via one-hot encoding for polymers (i.e., no chemical or structural descriptors) due to the small sample size of 28 distinct polymer structures tested.^40^ This encoding scheme transforms each polymer name into a binary vector of length 30, where each column corresponds to a specific polymer. The presence of a polymer is indicated by a value of 1 in the respective column, while its absence is denoted by a value of 0. For example, chitosan would be represented as (1, 0, 0,···, 0), whereas poly(ethylene oxide) (PEO) would be encoded as (0, 1, 0,···, 0). For solvents, we considered four different molecular fingerprinting schemes and assessed the predictive power of these schemes for solvents in conjunction with the one-hot encoding of polymer names. We finally selected the Morgan fingerprinting method for the solvent. Our analysis of the results of all the four solvent fingerprint schemes is described in the Supporting Information Section D. The inputs to our models comprise temperature, concentration (in mg/mL, with discrete values such as 5, 10, 50, etc.), one-hot polymer encoding, and solvent molecular features.

For modeling, we employed one of three ML architectures: a random forest (RF) regressor, XGBoost (XGB) regressor, and neural network (NN). While initially analyzing the impact of features on modeling, we solely used the XGB architecture due to its superior training speeds. Afterward, we compared each architecture against the Morgan solvent fingerprints. During the testing of the architectures, five folds were created by using a GroupKFold split based on polymer–solvent-concentration groupings. For the RF and XGB models, no scaling of features or transmission was done, and hyperparameters were tuned for the RF and XGB models using Scikit-Learn’s randomized search cv for 100 iterations with the five-folds.^41^ For the NN, both the training features and the target variables were initially scaled using a MinMaxScaler. Subsequently, the hyperparameters were fine-tuned through the Hyperband optimization technique, available in the KerasTuner Python package, leveraging an 80–20 split for training and validation data sets.^42,43^ The list of hyperparameters tuned and the set of values are provided in Supporting Information Table S4 and the optimal values are listed in Table S5.

During the model selection process, we identified an important challenge in using our transmission data. The data is heavily skewed toward 0 and 100% transmission (Figure 2d) and thus the resulting hyperparameters and optimization schemes were heavily influenced by these extremes of transmission. This can lead to poor predictions with the model overfitting to these regions and incorrectly showing lower error rates than realistically expected. For instance, an analysis of the polymer polytetrahydrofuran (PTHF) across 18 solvents revealed that all exhibited a transmission rate above 90%. This trend suggests models could infer that all solvents mixed with PTHF generally yield a transmission rate near 100%, with high accuracies, while, in reality, there are solvents such as isopropanol (IPA) that can precipitate at 10 °C. Thus, to select and set up the model to be less heavily influenced by extreme values of the transmission, we selected only data points with transmission percents within the 5–95% range when evaluating the models and tuning the hyperparameters. For the actual training of the models, we used the full range of data (0–100%) to enable comprehensive curve prediction and to preserve the important data in the extreme ranges.

Following the identification of optimal hyperparameters, we compared the model architectures using paired *t* tests with the goal of identifying the best performing architecture for subsequent experiments. By separating hyperparameter optimization (via cross-validation) from model architecture comparison (via paired *t* tests), we ensured a robust evaluation framework. For model architecture comparison, we created 30 random 50–50 train/test splits of unique polymer–solvent-concentration groupings, with the test splits only containing data between 5 and 95% transmission. We trained 30 models to evaluate their performance across the various data set partitions to run a paired *t* test to assess the statistical significance of the features and architectures tested. We chose these random train/test splits instead of the 5-fold splits because a *t* test requires the samples to be independent of one another, which is not the case for cross-validation data.^44^ This analysis will enable us to select which model, XGB, RF, and NN, provides the best performance in predicting transmission versus temperature data for samples not seen in the training data set.

## 3 Results

**Figure 3:**
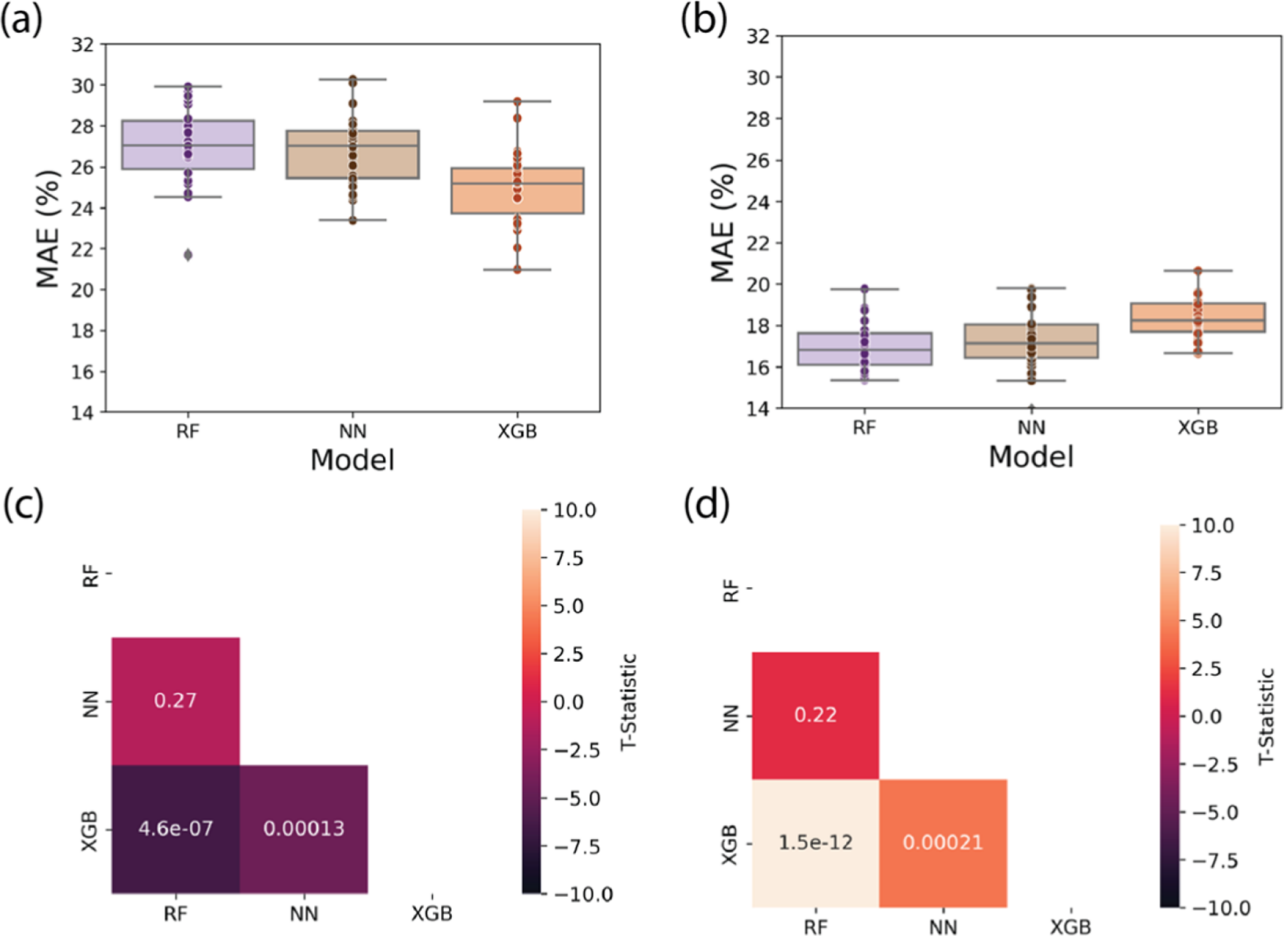
Box and whisker plots comparing the performance of RF, NN, and XGB models (a) when only data from 5 to 95% transmission was included in the data set (b) when test data situated at transmission levels below 5% and above 95% is included. Each individual dot represents the test score from one of the 30 50/50 train-test splits. Additionally, heatmaps display the T-statistic calculated from pair tests of different fingerprints (c) when only data from 5 to 95% transmission was included (d) when test data at transmission levels below 5% and above 95% was also used. Annotations within the heatmaps correspond to the *P*-values derived from the pair tests.

We first compared the XGB, RF, and NN models. When only data from 5 to 95% transmission was included in the data set, XGB demonstrated the best performance, followed by the NN and RF, which had comparable MAE values, as depicted in Figure 3a. We note that the magnitude of the MAE is high, but this is due to the use of a 50/50 split for model testing, and thus corresponds to testing the model’s ability to extrapolate with a low amount of training data. The superior performance of the XGB model may be due to its proficiency in handling tabular data compared to the other models and its resistance to overfitting data.^45^ The optimal architecture for each model is provided in Table S5. Notably, when we retained the test data situated at transmission levels below 5% and above 95%, the random forest and neural network models displayed similar performance, while the XGB model performed worse than them, as depicted in Figure 3b. Although the MAE is lower for the models run when the data below 5 and above 95% transmission is included than when it is excluded, this discrepancy is likely a result of overfitting to these extreme regions, rather than indicative of superior model architecture. Based on these results, for our production model, we use the XGB architecture with the hyperparameters tuned only with 5–95% transmission values, and with the Morgan fingerprint for the solvent.

**Figure 4:**
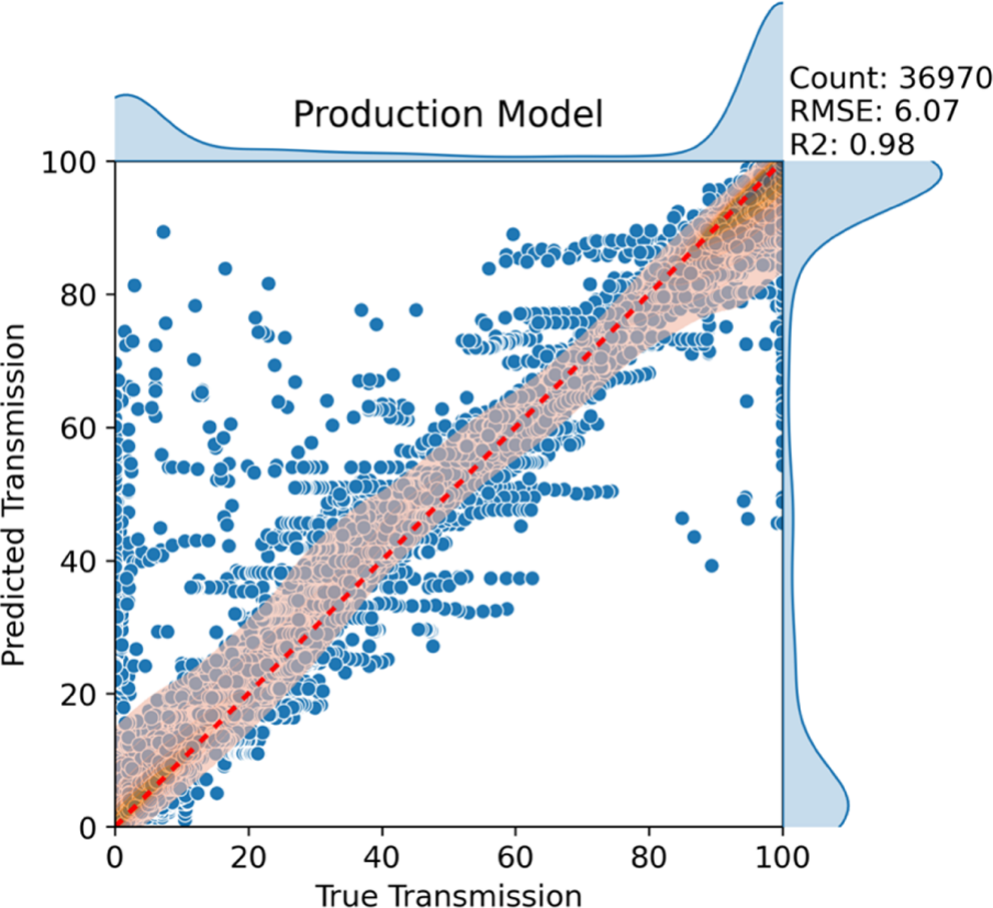
Production XGB model trained on Morgan fingerprints. The orange regions represent 2 day kernel density estimate (KDE) plots to showcase where most of the data is located. The darker the orange color, the more data there is. The side plots are 1 day KDE plots of true transmission (top) and predicted transmission (right).

We trained the production model on all our curated data (inclusive of <5 and >95% transmission data), with the performance of the model on the data displayed in the parity plot of Figure 4. The model achieved very good performance as indicated by the RMSE of 6% and *R*^2^ of 0.98. This indicates the model is not underfitting the data, as it is capable of accounting for 98% of the variance in the data. The low RMSE (6%) suggests that the predictions are reasonably accurate, which lends evidence to the assumption that the model is well-suited to the data. It is also clear from Figure 4 that a large proportion of the data falls in the extremes of transmission percent (below 5% and above 95%). Although it is not the focus of this work, we used XGB’s built-in method to analyze the importance of various features, keeping in mind that the choice of one-hot encoding for the polymer limits the feature insights available. We discuss this analysis in the Supporting Information Section E.

**Figure 5:**
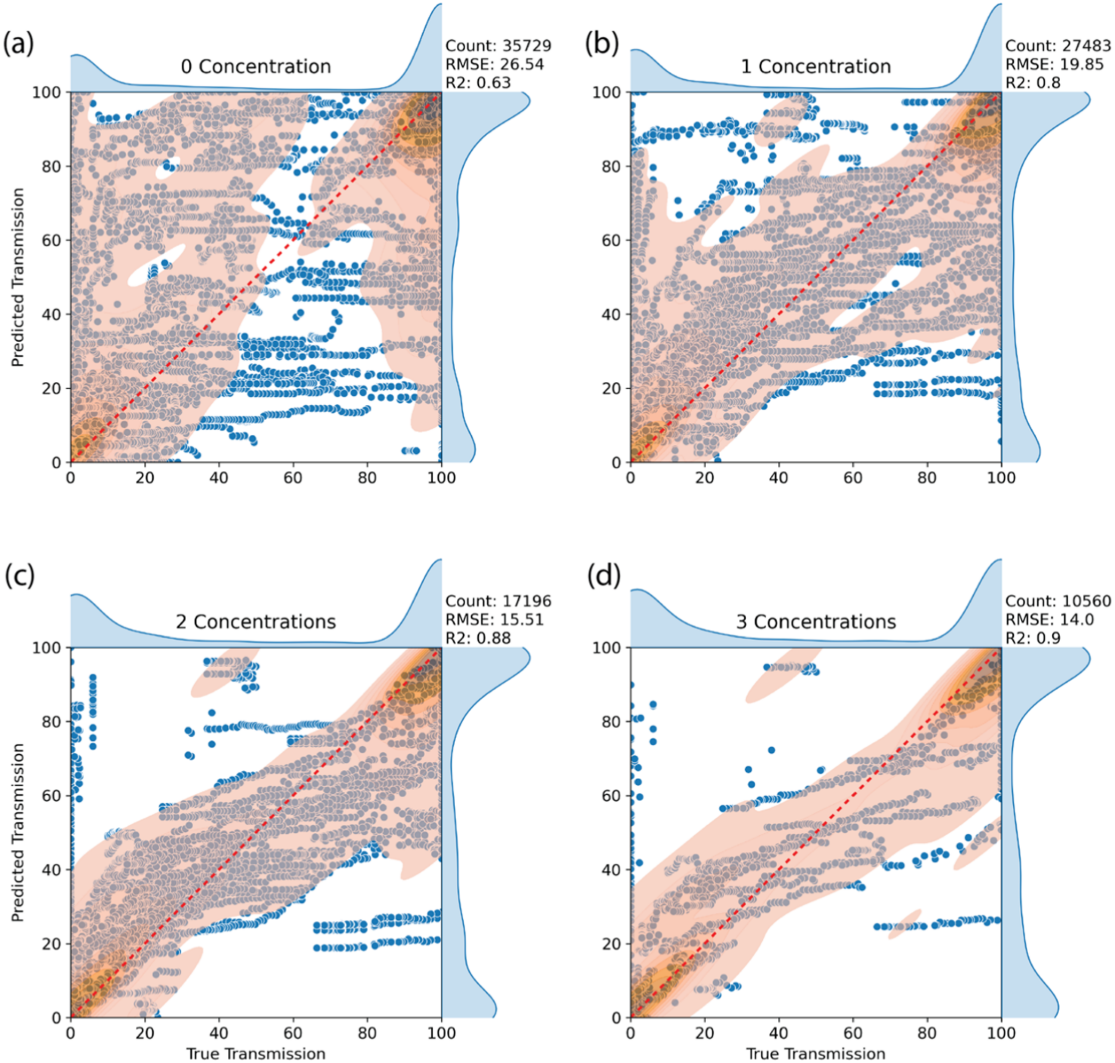
Parity plots of XGBoost model performance with leave-one-out (LOO) analysis on Morgan fingerprints. The orange regions depict 2-D kernel density estimate (KDE) plots, highlighting data distribution. Darker shades indicate denser data clusters. Accompanying side plots feature 1-D KDE plots of true transmission (top) and predicted transmission (right). Panels (a) through (d) illustrate model performance as increasing instances of polymer–solvent combinations are observed: (a) no prior instances, (b) one instance, (c) two instances, and (d) three instances.

To evaluate the model’s performance under conditions where the data has not been seen, we conducted a leave-one-out (LOO) analysis, categorized by polymer–solvent-concentration groupings. Referring to Figure 1, this process involves selecting a cell that represents the polymer and solvent to be tested. Initially, we train the model on all data except for the data in this cell, evaluating its performance across the different concentrations. Subsequently, we introduce one concentration from this cell into the training data, gauging its performance on the remaining held-out concentrations, followed by the introduction of two, and finally three additional concentrations. This process continued over every combination of concentrations present for the specific polymer–solvent pair. This test offers insights into the model’s proficiency in dealing with novel polymer–solvent pairings and how the model proficiency is impacted as different concentrations of a polymer–solvent pair are added to the test data. While our analysis focuses on XGB’s overfitting propensity, we recognize the importance of evaluating overfitting across all models. However, due to computational constraints, we relied on paired *t* tests to assess model performance for the other considered architectures (NN and RF). The outcomes are depicted in Figure 5, where panel (a) represents scenarios where the polymer–solvent group had not been encountered previously, while (b–d) illustrate the model’s performance when one, two, or three different concentrations of the polymer–solvent pair had been added, respectively.

Unsurprisingly, the model exhibits its poorest performance when encountering a polymer–solvent combination it has never encountered before, which is especially evident in transmission regions where values fall between 5 and 95%. However, even under such circumstances, the model manages to achieve a R^2^ score of 0.63, which is not uncommon for modeling polymer properties with this size of a data set.^40,46^ This moderate performance stems from the model’s ability to distinguish between scenarios of total insolubility (transmission of 0%) or solubility (transmission of 100%), albeit without precisely predicting transmission as it transitions between these states, as can be seen by the significant cluster of points on the parity line near 0 and 100% transmission in Figure 5a marked with darker orange.

The addition of even a single concentration generally enhances the model’s predictive capabilities, especially in intermediary transmission ranges. This can be seen by the convergence of the 2-D KDE plot toward the parity line in Figure 5b and the increase of the *R*^2^ score to 0.8. Expanding the data set to include two and three concentrations yields even greater performance enhancements as indicated by the RMSE and *R*^2^ and further convergence of the 2-D KDE plot toward the parity line in Figure 5c,d. With three concentrations included, the *R*^2^ rises to 0.9 and RMSE decreases to 14.02. This indicates that the model’s ability to predict the percent transmission for a given polymer/solvent pair at a given concentration improves as the model sees other concentrations of that polymer/solvent pair, but that at 3 concentrations it already has quite good performance. This knowledge can guide data set development to optimize the required number of experiments needed to build a valuable data set. Each experiment takes approximately 13.5 h, so the ability to make good predictions with 3 concentrations (rather than 4, 5, etc.) can save significant resources.

**Figure 6:**
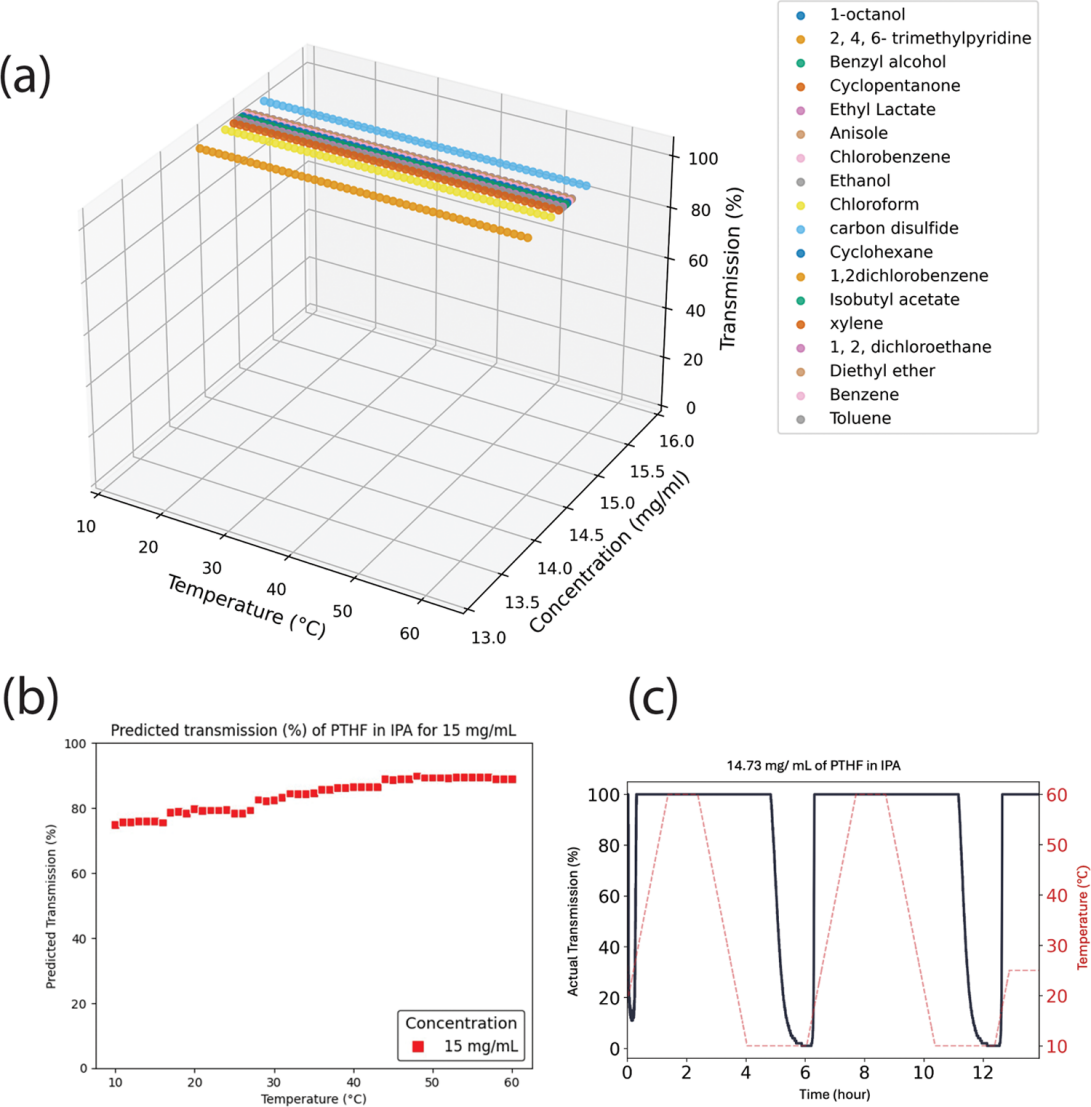
(a) Transmission vs temperature data for PTHF in different solvents in the database, which demonstrates that PTHF in soluble in all 18 solvents tested. (b) shows predicted transmission (%) of PTHF in isopropyl alcohol (IPA) for 15 mg/mL. (c) shows the collected results of Crystal16 on PTHF in IPA, with the experimental validation confirming that 14.73 mg/mL of PTHF precipitates in IPA at 10 °C.

Despite the promising performance of the model, the overly large number of the transmission data at <5% and >95% transmission is expected to be problematic for some polymers and solvents. Here we examine the case where the data for a polymer or a solvent always falls either at <5% or at >95% transmission and the implication on the ability of the model to extrapolate. In all tested solvents, two polymers showed distinct transmission values: poly(ethylene terephthalate) (PET) always exhibited values below 10%, while PTHF always showed complete transmission at 100%. Our hypothesis is that the production model will fail to identify whether this behavior differs for these polymers in other solvents and it will continue to report the fully soluble and insoluble transmission values for these polymers regardless of the solvent. We demonstrate that this hypothesis is accurate for PTHF, as illustrated in Figure 6. We found that the transmission is 100% for PTHF in the 18 solvents tested and used for training data (Figure 6a). To test the hypothesis, we predicted the transmission versus temperature for isopropyl alcohol (IPA), a solvent not in the data set, with the results shown in Figure 6b. The plot shows that the predicted transmission (%) is 75% at 10 °C and reaches 95% at 60 °C. However, experimental data for PTHF in IPA shows that the transmission drops to 0% at 10 °C, Figure 6c, demonstrating that the model was unable to accurately predict the transmission for this polymer/solvent pair. These results confirm that a polymer/solvent that exhibits consistently soluble or insoluble behavior in the data set may lead to inaccurate predictions for unseen solvents, as the data is tailored to the extremes of <5% and >95%.

So far, we have analyzed the quality of prediction of the experimental transmission vs temperature data. The motivation for predicting this data, rather than predicting quantities that more directly capture solubility, such as the phase diagram or Hansen solubility parameters, or classifying polymer–solvent pairs into “soluble” or “insoluble” is that the transmission data set can be used by many practitioners to predict the solubility quantities of interest to their application. We present an example of such a use here by converting the true and predicted values of transmission into three classes. Pairs were labeled “insoluble” when transmission was <10%, “soluble” when transmission was >85% and “partially soluble” when transmission was between 10 and 85% inclusive. Although we use the terminology of “insoluble” and “soluble” we are referring to nonequilibrium solubility during the cooling ramp, albeit under a consistent cooling ramp for all tests, 0.5 °C/min, which is significantly improved compared to prior data sets that do not report and consistently control these parameters. As we grow the data set we will include more equilibrium hold times and enable predictions of equilibrium solubility, but the focus here is to demonstrate the modeling approach with this data set measured under precise conditions.

**Figure 7:**
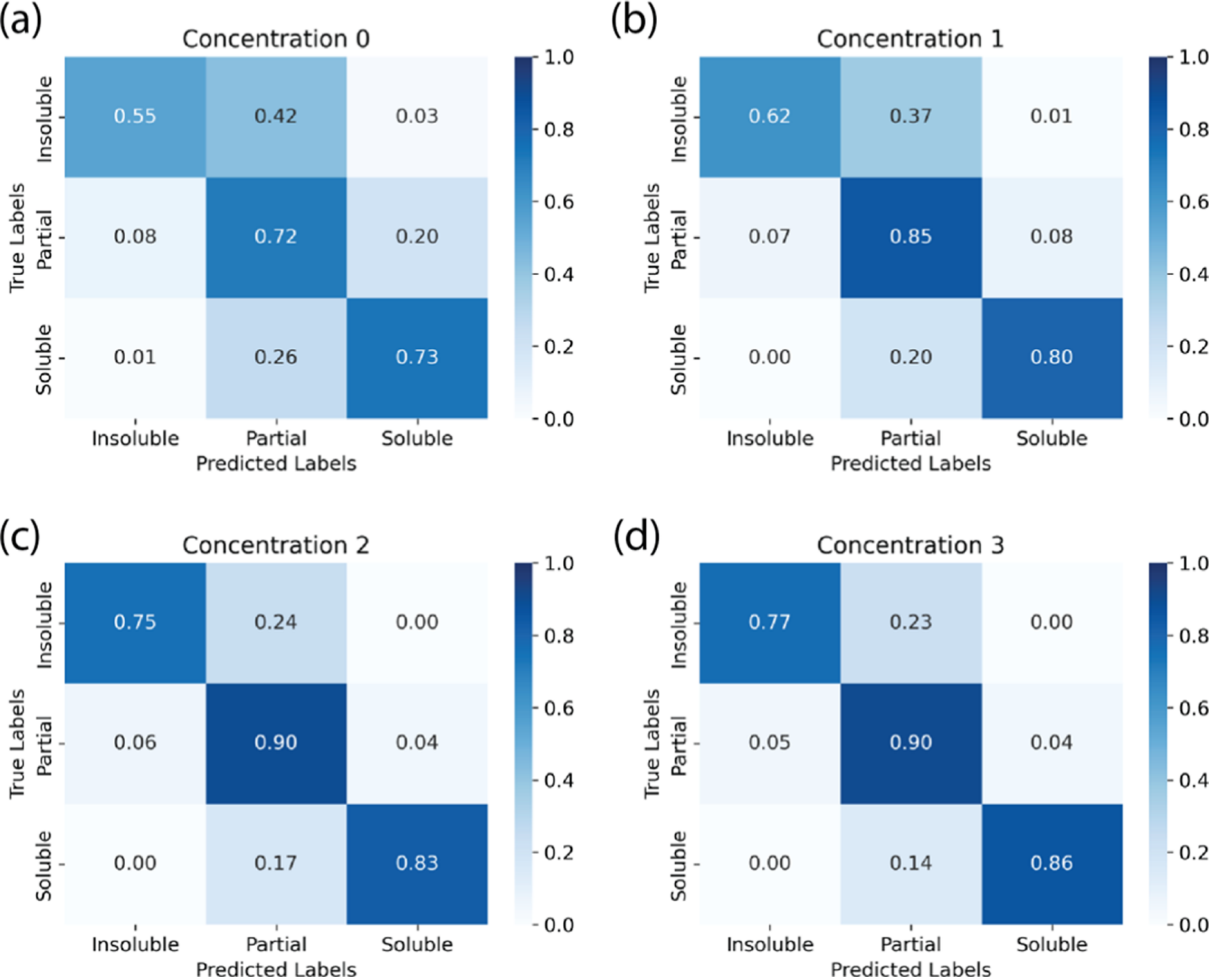
Confusion matrix illustrating the classification performance of the data depicted in Figure 5. The regression data has been classified into three categories: “Insoluble” (transmission < 10%), “Soluble” (transmission > 85%), and “Partial” (transmission between 10 and 85% inclusive). “Panels (a) through (d) illustrate model performance as increasing instances of polymer–solvent combinations are observed: (a) no prior instances, (b) one instance, (c) two instances, and (d) three instances.

Figure 7 shows the confusion matrix of the data as if it were a classifier instead of a regressor, based on the three classes defined above. In Figure 7a, where unique polymer–solvent pairs are being tested, we see that the model is very good at differentiating soluble and insoluble, as indicated by the low numbers in the upper right and lower left corners. However, the model tends to be incorrect when making a partially soluble prediction, as indicated by the high counts in the central top and bottle cells. As further concentrations are added to the training data (Figure 7a–d increases in number of concentrations per polymer/solvent pair in the data set), the prediction of all three classes improves, though partial solubility is still relatively poor, even with 3 concentrations included in the training data. This is likely due to the low number of polymer/solvent/concentration combinations that had any data in the window from 10 to 85% transmission (131 unique polymer–solvent-concentration combinations). However, it can also be due to the nonequilibrium nature of some of the data in the partial solubility category, which is more likely to have nonequilibrium data than the fully soluble or fully insoluble category. These kinetics considerations are the subject of future work with this data set.

**Figure 8:**
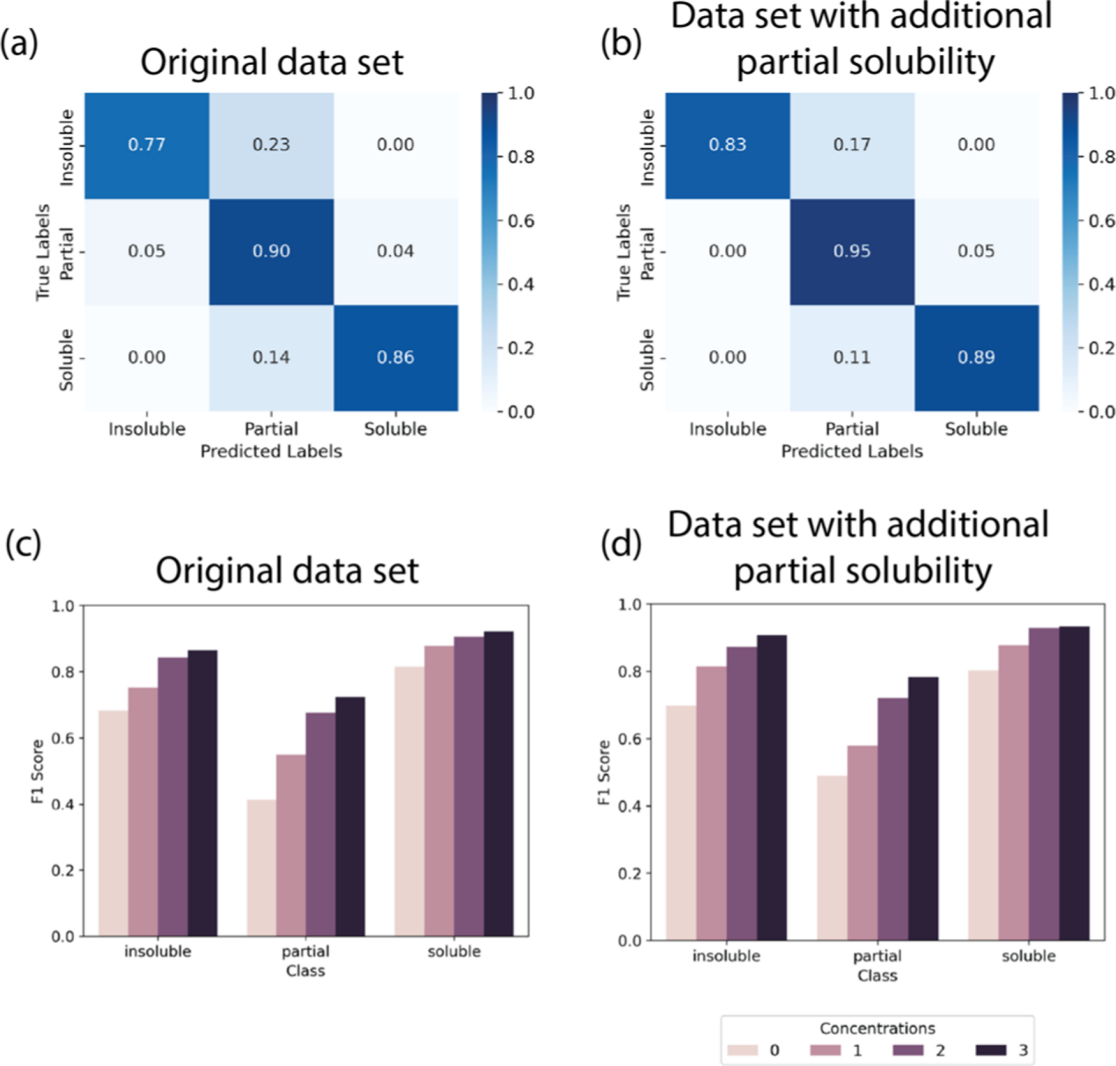
(a) Confusion matrix for 3 instances of concentrations seen for each polymer/solvent pair illustrating the classification performance of the data depicted in Figure 5 and (b) confusion matrix for 3 instances of concentrations seen for each polymer/solvent pair illustrating the classification performance of the data with additional targeted partial solubility data. The regression data has been classified into three categories: “Insoluble” (transmission < 10%), “Soluble” (transmission > 85%), and “Partial” (transmission between 10 and 85% inclusive). (c) F1 scores for classification performance of data depicted in Figure 5 and (d) F1 scores for classification performance with additional targeted partial solubility data.

Given that the predictions for partial solubility were poor relative to insoluble and soluble and that this was due partially to a low amount of data, we independently collected targeted data for polymer/solvent pairs that demonstrated partial solubility and included them in the training data set. This provided 44 additional polymer–solvent-concentration points for the partial solubility range. We compared the performance using the confusion matrix and F1 score and show the confusion matrix results for 3 concentrations in training and F1 score in Figure 8. The confusion matrices for all concentration instances with the new data are shown in Supporting Information Figure S10. The ability to correctly classify partial solubility was significantly enhanced by the addition of more data in that range, as seen by the lower values in the central top and bottom cells in Figure 8a and b compared to (a). Additionally, the F1 score was higher for all concentrations in all classes, especially the partial solubility class, showing improved model performance with respect to precision and recall.

Comparing the F1 scores to a prior binary classification model, the F1 scores in this work were ∼0.6–0.7 for unique polymer–solvent combinations (those not seen in training data) and prior work F1 scores were 0.8–0.9.^22^ While this shows that the performance of the predictions here was lower than prior work, there are key differences. The data set size is much smaller and less diverse than the prior work (which has ∼ thousands of polymers and dozens of solvents). The current study’s high-fidelity data does surpass the previous data set quality, which sometimes contained contradictory solubility labels, and while this can be a strength eventually, it requires significant time investment to generate the data set leading to lower number of points. Overall, this analysis demonstrates that a classification based on true and predicted values of transmission percent performs well for soluble and insoluble categories, where the data set contains many data points, but that targeted additional experiments in the partial solubility range can improve predictions and enable this higher level of granularity in solubility classification compared to binary predictions performed previously.^22,24^

Moving beyond binary classification of solubility for polymer/solvent pairs to this three level description with data at different temperatures and concentrations has important implications for how the ML predictions may be used by practitioners. Solid/liquid equilibrium results in polymers being soluble at some temperatures, but not others and only being soluble up to a specific concentration. Predicting full phase diagrams for polymer/solvent pairs is one way to incorporate this information,^25,47^ but it is time-consuming to do for a broad chemical space and unnecessary for helping to guide formulators and process designers. Instead, having information at common operating temperatures (such as 10–60 °C used here) provides guidance relevant to industrial use. Incorporating predictions for partial solubility and multiple known concentrations provides guidance that a practitioner may use to judge that the polymer may be soluble at slightly lower concentrations or different temperatures, allowing a user to extrapolate with fewer trial-and-error experiments than with only binary classes without concentration information, as done previously with classification models. However, to our knowledge, there are no rigorous studies quantitatively identifying what a “useful” level of solubility prediction would be, a weakness in assessing value of prediction tools. As shown here, with the small, controlled data set we can achieve success in some predictions and further data collection in the partial solubility range can improve predictions and could eventually lead to even more classes and greater granularity.

## 4 Conclusions

This work serves two primary purposes. The first is to provide a high quality data set that can be a resource for researchers to analyze and model solubility relationships and the second is to demonstrate modeling applications and challenges with this data set, illustrating the potential for machine learning models to predict solubility with well-controlled turbidity data sets while highlighting caveats such as overtraining on extreme values. This study demonstrates how experimental transmission data collected through a turbidity method, in conjunction with machine learning models, can be effectively used to forecast the experimental transmission data for polymer–solvent combinations. Experimental data was collected using the Crystal16 parallel crystallizer in a high throughput manner. The model training was done for different unique polymer–solvent combinations varying in temperature and concentration. Precise predictions were attained by studying the % transmission vs temperature by leveraging the XGBoost model in combination with one-hot encoding and Morgan fingerprinting. The model achieved good performance, with a RMSE of 6% and a coefficient of determination (*R*^2^) of 0.98. The current database is more tailored to completely soluble and insoluble cases with transmissions of 100 or 0%, respectively, so it can generate good prediction for cases on the two ends of the spectrum. The model’s performance was assessed using a LOO analysis on polymer–solvent concentration groupings, where the data was not previously observed and a clear improvement in the predictions was seen as more concentrations for each polymer/solvent pair were added, but good performance was seen with only 2–3 concentrations per pair.

We extended the work beyond transmission predictions to analyze the performance if we use the predicted transmission data to classify into “insoluble”, “partially soluble”, and “soluble”, although the data set used for training is not always at equilibrium, so these are referring to the phase behavior during cooling at 0.5 °C/min. This adds a level of granularity in solubility prediction compared to prior work, which only classified as solvent/nonsolvent. We found good prediction for soluble/insoluble, with less accuracy for the partially soluble case due to the low number of data points and higher likelihood of nonequilibrium values for partial solubility. Targeted addition of more partial solubility data further increased those predictions, however, showing a path to using the turbidity data for practical solubility predictions. With this promising starting point for prediction of experimental data that can be translated to practical solubility information, further data collection on polymers with varying solubility classes can enhance the model’s predictive capabilities across a broader range of solvents and polymers and an in-depth analysis of the kinetics will enhance understanding of the limits of the data in predicting equilibrium solubility. Overall, the model’s ability to predict transmission percentage highlights its potential for industrial applications where the prediction of the solution behavior of the polymer across different temperatures is important.

## Data Availability

Data available on the Github repository: https://github.com/mona2442/Polymer-Solubility-Data_Crystal16

## Supporting Information Available

list of polymers and solvents used to create the database; examples of unequilibrated and high variation raw and filtered data; analysis of data removed during processing; discussion of fingerprinting selection for solvent; feature analysis; and the confusion matrix evaluation of model run with additional partial solubility data is included in supporting information-ML models turbidity and solubility (PDF)
SMILES strings for the polymers are provided in polymers_smiles (XLSX)
SMILES strings for the solvents are provided in solvents_smiles (XLSX)
