# Supplementary material for: Machine Learning Models for Predicting Polymer Solubility in Solvents across Concentrations and Temperatures
Source: J Phys Chem B. 2024 Dec 12;128(51):12786–97. doi: 10.1021/acs.jpcb.4c06500 (PMC11684024; doi:10.1021/acs.jpcb.4c06500)
Supplement: Supplementary file 1 — jp4c06500_si_001.pdf [file jp4c06500_si_001.pdf]

Supplementary Information

Machine Learning Models for Predicting Polymer Solubility in Solvents Across Concentrations and Temperatures

Authors: Mona Amrihesari,[1^] Joseph Kern,[2^] Hilary Present,[3] Sofia Moreno Briceno,[1] Rampi Ramprasad,[2] Blair Brettmann[1,2] *

*These authors contributed equally
*Corresponding author, blair.brettmann@chbe.gatech.edu

1) School of Chemical and Biomolecular Engineering, Georgia Institute of Technology, Atlanta, GA 30332
2) School of Materials Science and Engineering, Georgia Institute of Technology, Atlanta, GA 30332
3) School of Computational Science and Engineering, Georgia Institute of Technology, Atlanta, GA 30332

A. Polymers and solvents used to create the database

Table S1:shows the list of polymers have been used for solubility model training. Molecular weight, PDI, manufacturer and the CAS numbers when available recorded as follows. Mw_min and Mw_max recorded when there is a range for molecular weights. If there is only one number, then the Mw_min and Mw_max is equal. ID is related to internal ID system for extractions of the raw data and link that for modeling. The IDs are skipped because polymers that has not been tested so far, are removed from this table.

ID	name	Acronym	Mw_min	Mw_max	Mn	PDI	supplier	CAS
31	Poly Styrene	PS	10000	10000			Sigma-Aldrich	9003-53-6
32	Polyvinylpyrrolidone	PVP	10000	10000			Sigma-Aldrich	9003-39-8
33	Poly methyl methacrylate	PMMA	15000	15000			Sigma-Aldrich	9011-14-7
34	Poly ethylene glycol	PEG	8000	8000			Sigma-Aldrich	25322-68-3
35	Polyvinyl alcohol	PVA	9000	10000			Sigma-Aldrich	9002-89-5
36	Polycaprolactone	PCL	14000	14000			Sigma-Aldrich	24980-41-4
37	Polypropylene	PP	15000	15000			Sigma-Aldrich	9003-07-0
38	Poly L lactic acid	PLLA	5000	5000			Sigma-Aldrich	26100-51-6
39	Dextran	Dex	9000	10000			Sigma-Aldrich	9004-54-0
40	Poly(2-ethyl-2-oxazoline)	PEOX	5000		5000	<=1.3	Sigma-Aldrich	25805-17-8
41	Poly4vinylphenol	P4VP	9000	11000			Polysciences	25232-41-1
42	Chitosan	Chi	15000	15000			Polysciences	9012-76-4
43	Nylon-6	Nylon-6	35000	35000			Polysciences	25038-54-4
46	Poly ethylene oxide	PEO	1000000	1000000			Alfa Aesar	25322-68-3
48	Poly (vinylidene fluoride)	PVDF	534000	534000			Sigma-Aldrich	24937-79-9

53	Nylon-12	Nylon-12	100000				Sigma-Aldrich	24937-16-4
54	Polyethylene terephthalate	PET	10000				Sigma-Aldrich	25038-59-9
57	Poly(tetrafluoroethylene)	PTFE					Sigma	9002-84-0
58	Poly ethylene	PE					Thermo Fisher scientific	9002-88-4
59	Polyvinyl alcohol	PVA	145000	180000			Sigma-Aldrich	9002-89-5
60	Polyvinylpyrrolidone	PVP	1300000	1300000			Sigma-Aldrich	9003-39-8
61	Polyacrylamide	PAM	40000		40000		Sigma-Aldrich	9003-05-8
62	Poly(ethylene glycol) methyl ether	mPEG	5000		5000		Sigma-Aldrich	9004-74-4
63	Polyacrylonitrile	PAN	150000				Sigma-Aldrich	25014-41-9
64	polytetrahydrofuran	PTHF	2900	2900			Sigma-Aldrich	25190-06-1
65	Cellulose acetate		50000	50000			Sigma-Aldrich	9004-35-7
66	Poly(vinylamine) hydrochloride		25000	25000			Polysciences	26336-38-9
68	Polyvinyl chloride	PVC					Sigma-Aldrich	9002-86-2
69	Polyethyleneimine-linear		2100	2100			Sigma-Aldrich	9002-98-6
70	Poly(vinyl formal)						Sigma-Aldrich	9003-33-2
71	polyacenaphthylene		10000	10000			Sigma-Aldrich	25036-01-5
72	Poly(chlorotrifluoroethylene)						Sigma-Aldrich	9002-83-9

Table S2: Shows the list of solvents in our dataset and their ID, percent of its purity, supplier and CAS numbers. The IDs are skipped because solvents that has not been tested so far, are removed from this table.

ID	name	percent purity	supplier	CAS
1	Water	NULL	N/A	7732-18-5
2	m-cresol	99	Acros Organics	108-39-4
3	Methanol	99.9	Sigma-Aldrich	67-56-1
4	Ethanol	99.5	Fisher bioreagents	64-17-5
5	Isopropylalcohol	99.9	Sigma-Aldrich	67-63-0
6	n-butanol	99.4	Thermo Fisher scientific	71-36-3
8	2,2,2-Trifluoroethanol	99.8	Acros Organics	75-89-8
9	Nitrobenzene	99	Thermo scientific	98-95-3
10	Chlorobenzene	99	Honeywell	108-90-7
11	NMP	99	Acros Organics	872-50-4
12	DMAc	99	Thermo Fisher scientific	127-19-5
13	DMSO	99.9	Thermo Fisher scientific	67-68-5
14	DMF	99.8	Beantown Chemical	68-12-2
15	Acetonitrile	99.5	VWR Chemical	75-05-8
16	Acetone	99.5	VWR Chemical	67-64-1
17	DCM	NULL	Anachemia	75-09-2
18	THF	99	Sigma-Aldrich	109-99-9
19	Ethyl Acetate	99.5	VWR Chemicals	141-78-6
20	Cyclohexanone	99.8	Acros Organics	108-94-1
21	Formamide	Ultra pure	VWR Life science	75-12-7
22	1,2,4-trichlorobenzene	99	Sigma-Aldrich	120-82-1
23	1,4-dioxane	99.8	Thermo Fisher scientific	123-91-1

24	Chloroform	99.8	Termo Scientific	123-91-1
25	Diethyl ether	99.8	Sigma-Aldrich	67-66-3
26	Toluene	99.8	Sigma-Aldrich	108-88-3
27	Benzene	99	Sigma-Aldrich	71-43-2
28	n-hexane	99	Sigma-Aldrich	110-54-3
29	Petroleum ether	NULL	Ward's science	8032-32-4
30	carbon disulfide	99.9	OmniSolv	75-15-0
31	Heptane	99	VWR Chemicals	142-82-5
32	Cyclohexane	99.5	TCI AMERICA	110-82-7
34	n-Pentane	98	Beantown Chemical	109-66-0
35	Ethyl Lactate	98	TCI AMERICA	97-64-3
36	styrene	99	Thermo Fisher scientific	100-42-5
37	1, 2- dichloroethane	99	OmniSolv	107-06-2
38	xylene	NULL	Leica	95-47-6
39	1,2- dichlorobenzene	99	Sigma-Aldrich	95-50-1
40	n-Butyl Acetate	99	Thermo Fisher scientific	123-86-4
41	Pyridine	99	Honeywell	110-81-1
42	Anisole	99	Beantown Chemical	100-66-3
44	2, 4, 6- trimethylpyridine	99	Sigma-Aldrich	108-75-8
45	Cyclopentanone	99	Sigma-Aldrich	120-92-3
46	Isobutyl acetate	99	TCI AMERICA	110-19-0
47	1-octanol	99	Sigma-Aldrich	111-87-5
48	Benzyl alcohol	99	Sigma-Aldrich	100-51-6

B. Examples of unequilibrated and high variation raw and filtered data

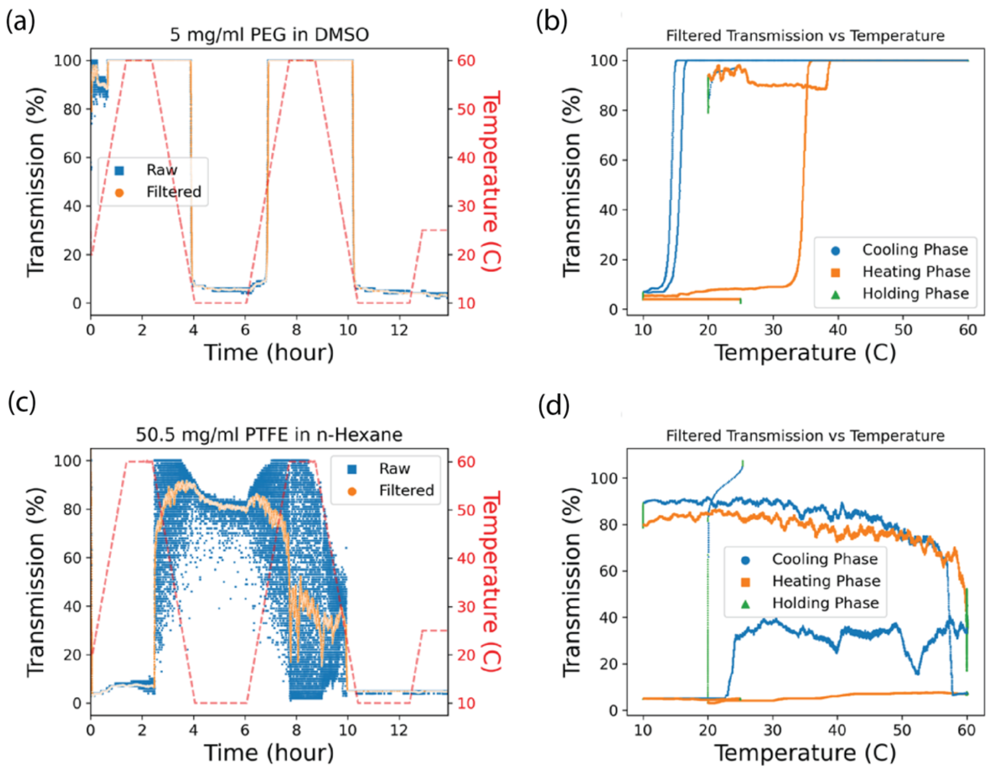

Figure S1. (a) Example of raw data that is unequilibrated in the first temperature ramp phase. (b) Filtered transmission data from (a) plotted against temperature. The variation in transmission vs temperature for the two heating phases is indicative of unequilibrated data at the start of the experiment. The low variability in the holing phases at 10 C and 60 C is indicative of a good behaving experiment otherwise. (c) Example of poorly behaving raw data. (d) Filtered data from (c) plotted against temperature. Large variations in transmission at same temperatures and phases is indicative of poorly behaving data, particularly for holding phases.

C. Analysis of data removed during data processing

We analyzed the number of polymer/solvent/concentration combinations prior to data processing and after data processing to check for bias in which samples were removed. The counts are shown in Figure S2 below.

There is not a strong bias in which concentrations were removed: 5 mg/mL decreased by 9%, 15 mg/mL decreased by 18%, 30 mg/mL decreased by 12% and 50 mg/mL decreased by 25%.

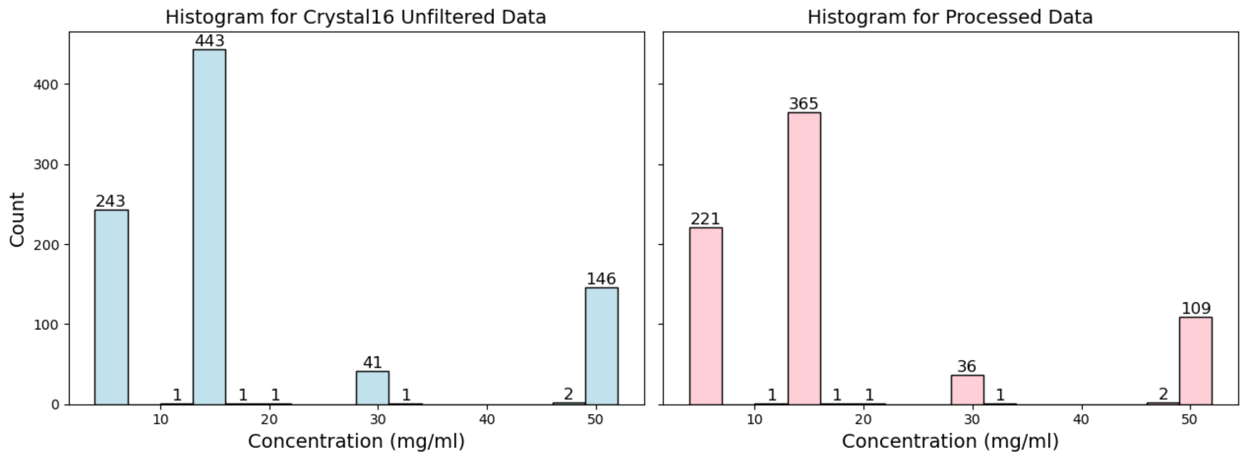

Figure S2: Counts of polymer/solvent pairs for each concentration in the dataset before and after data processing.

D. **Selection of a fingerprinting method for the solvent**

The four fingerprint methods tested for the solvent were one-hot encoding, Kurotani, Hansen and Morgan (Figure S3). The one-hot encoding transformed the set of 30 polymer samples and 45 solvents into a vector of length 75. A value of 1 was entered when the solvent was present and a value of 0 was entered when the solvent was not. The weakness of this fingerprint method is that the model can only learn associations between polymer-solvent pairs since features common to multiple polymers and solvents are not encoded into the vector. The fingerprinting method by Kurotani et al.[1] includes specific molecular features. The binary encodings indicate whether the molecule contains compositions such as nitrogen and sulfur, whether it has molecular features such as NH and OH and the number of aromatic rings, among other important features. We used this method with an added feature, MolLogP, which estimates the partition coefficient between n-octanol and water for the molecule being fingerprinted, offering insights into its solubility in oil and water [2]. Each of these features was extracted using the RDKit cheminformatics software [3]. Descriptors were extracted using the Chem.Descriptors.CalcMolDescriptors function, and compositions were extracted using simplified molecular-input line-entry system (SMILES) arbitrary target specification (SMARTS) encodings provided in Table S3. This fingerprinting method could be an improvement over the one-hot encoding since it enables analysis of how molecule features may impact the predicted outcomes.

(a) One-Hot

Polymer	Solvent
PE	DMSO
PE	DMF

↓

PE	DMSO	DMF
1	1	0
1	0	1

(b) Kurotani

Contains molecular compositions	N, S, halogens (Cl, Br, I, F), >CO, –OH, –CHO
Count of molecular compositions	C, H
Contains molecular descriptors	NHOHCount, NumAromaticRings, NumHAcceptors, NumHDonors, NumHeteroatoms, RingCount
Exact molecular descriptors	Chi0n, HallKierAlpha, MaxPartialCharge, MinPartialCharge, SlogP_VSA12, SMR_VSA5, SMR_VSA10, VSA_EState9
Additional	MolLogP (solvent), Longest sidechain (polymer)

(c) Hansen

dD	dH	dP
17.6	0.4	0.3
16.6	10.7	3.9
19.6	6.8	4

(d) Morgan

1	2	...	2048
1	0	...	0
0	1	...	1
0	1	...	0

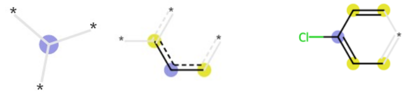

Figure S3. Schematic of different fingerprinting methods. (a) One-hot encoding of solvents by name. (b) Descriptors as defined in [1] plus an additional descriptor for solvents (MolLogP). (c) Hansen solubility parameters extracted from HSPiP. (d) 2048 bit vector Morgan fingerprint extracted using RDKit with a radius of 2.

The third fingerprint method was the Hansen solubility parameter approach extracted from the HSPiP software (Figure S3(c)) [4]. These parameters were chosen as they are currently the industry standard for estimating solubility. They are based on the idea that like molecules should dissolve like molecules, and encode hydrogen bonding (d_H), intermolecular (d_P), and dispersion (d_D) forces between molecules. Finally, we assessed a 2048-bit Morgan fingerprint with a radius of two [5], [6]. The radius denotes the maximum number of nearest neighbors of a central atom the algorithm explores. Initially, the algorithm catalogs the central atom and the number and type of non-hydrogen bonds it possesses, hashes this value, and converts a single bit in the 2048-bit fingerprint to 1 based on the corresponding hash bucket. This process ensures that the value remains 1 if it was already set. Subsequently, a graph is created from the central atom, including its first nearest neighbors, and the same procedure is repeated. This iterative process continues until the maximum radius is reached, in this instance, the second nearest neighbor graph, and repeats for all atoms, as illustrated in Figure S3(d). Any columns that were zero for all molecules were dropped, resulting a vector of length 184.

Table S3. SMARTS queries used to search for compositional features in the molecules during Kurotani fingerprinting.

Compositional Feature	SMARTS
C	C
N	N
S	S
Halogens	[Cl,Br,I,F]
>CO	C=O
-OH	[OH]
-CHO	[CH]=O
H	[H]

In our examination of solvent fingerprints, all models exhibited average mean absolute errors (MAE) exceeding 20%. This poor performance was expected, as the test predictions were conducted on unique polymer-solvent-concentration splits within transmission ranges where transmission data was relatively scarce. Other than the one-hot encoding for solvents, all other methods of fingerprinting the solvents achieved comparable results, as seen in Figure S4.

The subpar performance of the one-hot models can be attributed in part to their incapacity to generalize to unfamiliar solvents. In 22 out of 30 randomly generated 50/50 splits, the training dataset lacked exposure to 1 to 3 solvents present in the test dataset, which means the model would not be able to make a logical prediction on these solvents in the test data. However, despite a slight deterioration in the T-statistic and an elevation in the P-value upon the exclusion of these splits, as depicted in Figure S4, the superiority of the other three feature sets over the one-hot encoding persists. This suggests a correlation exists between transmission % and these other features that the model can learn from.

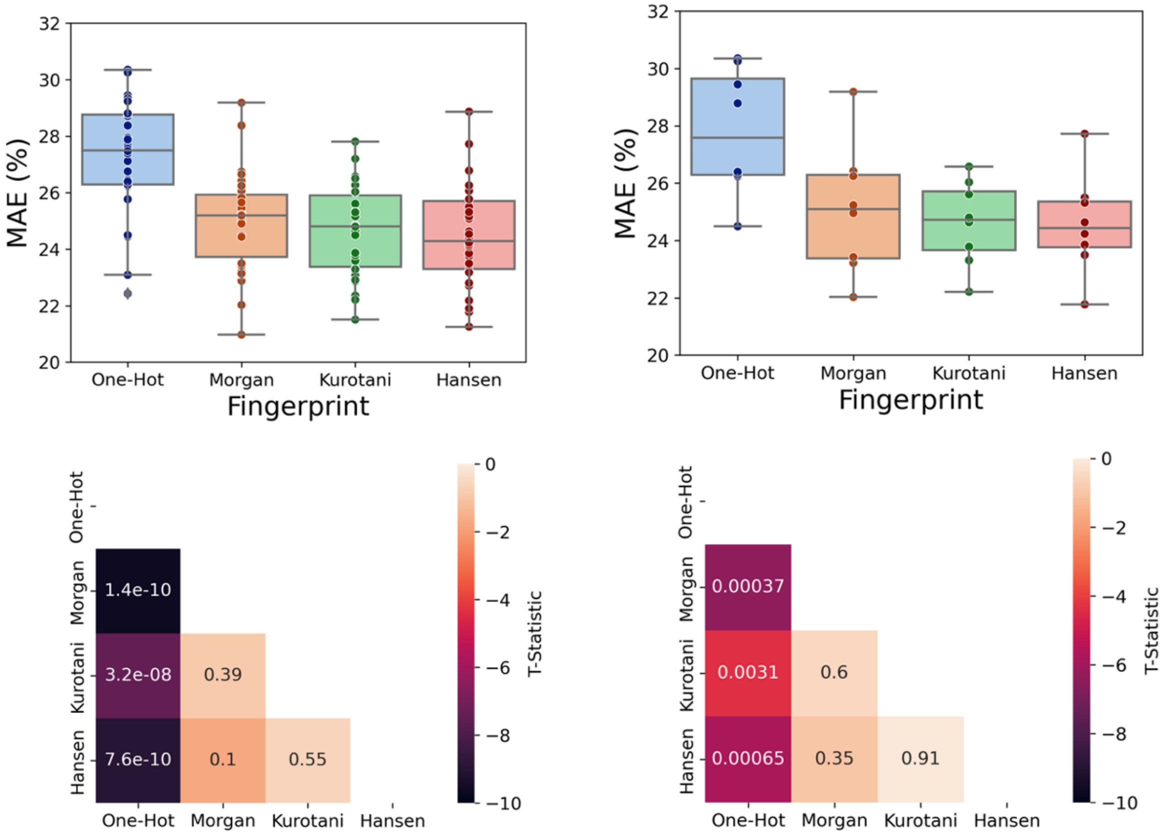

Figure S4. (a) Box and whisker plots comparing the performance of XGB model using different fingerprints. Each individual dot represents the test score from one of the 30 50/50 train-test splits (b) Box and whisker plots comparing the performance of XGB model using different. employing Morgan fingerprints. Each individual dot represents the test score from one of the 8 50/50 train-test splits. The 22 other splits were removed due to some solvents not existing in both the train and test set. Additionally, heatmaps display the T-statistic calculated from pair tests of different fingerprints (c) and different models (d). Annotations within the heatmaps correspond to the P-values derived from the pair tests.

The remaining three fingerprints exhibited statistically comparable performance, suggesting each can capture crucial molecular information related to solubility. This outcome likely stems from intercorrelations among the various feature sets, as illustrated in Figure S5. Hansen fingerprints encapsulate molecular dispersion forces (d_D), polar forces (d_P), and hydrogen bonding (d_H)—parameters pivotal in determining solubility [4], [7]. Often computed through functional group contribution methods, these features share commonalities with the Morgan fingerprint, which also characterizes molecular functional groups. It seems the model is adept at discerning the impact of these functional groups on solubility, capable of mapping such effects without necessitating transformation into the lower-dimensional Hansen space. For instance, bits 222, 730, 807, 1004, and 1410 are all correlated with the hydrogen bonding Hansen parameter. In Figure S6 all Morgan bits are displayed, and we see these groups correspond to entities containing alcohols, esters, and amides—all capable of hydrogen bonding. Similarly, many Kurotani features simplify to one-hot encodings of specific elements (e.g., N, S, Cl), functional groups (e.g., alcohol, aldehyde, aromatic rings),

or numerical representations of molecular characteristics. These features, as evidenced in Figure S5, are also captured by the Morgan features.

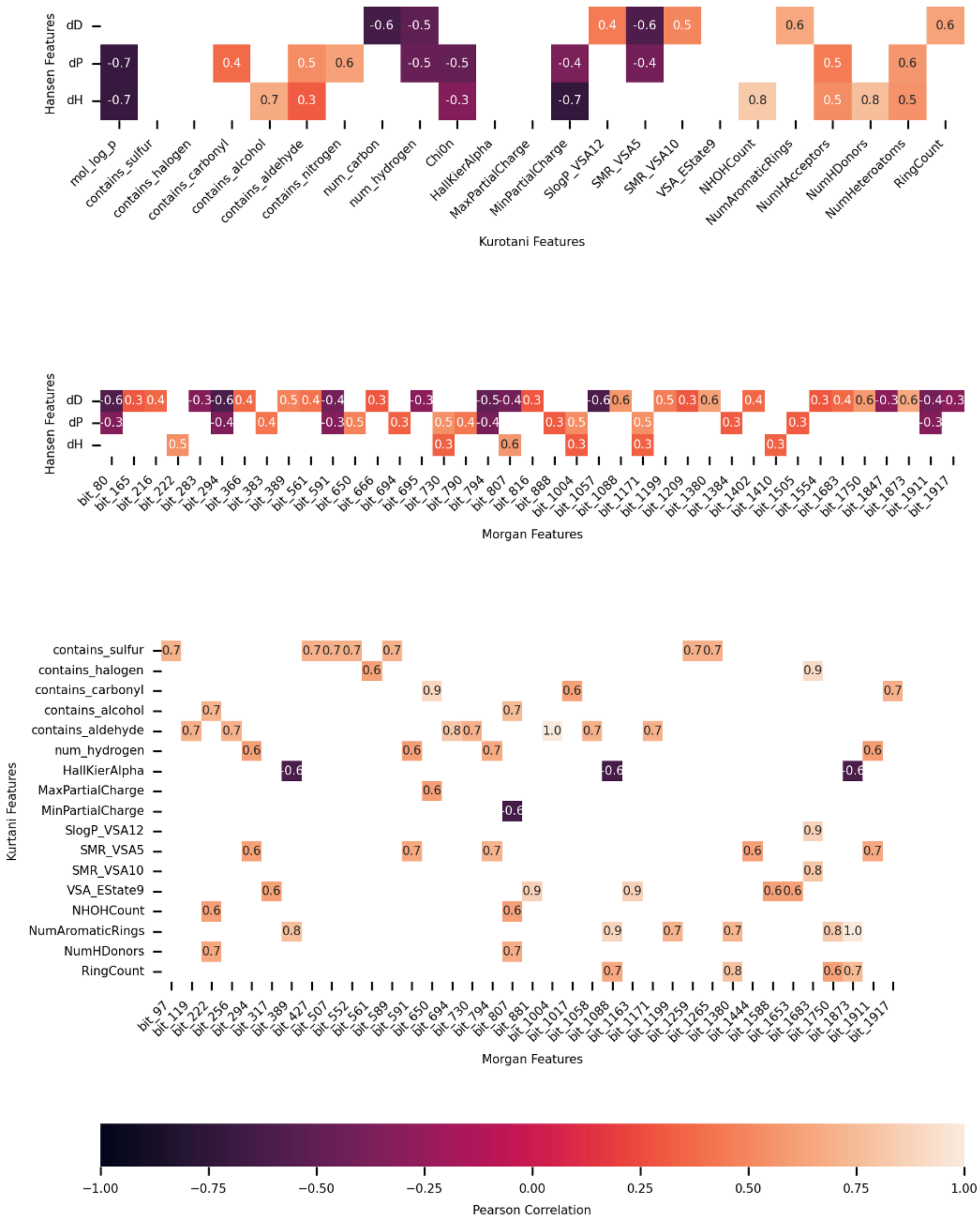

Figure S5. Pearson correlations between Hansen, Morgan, and Kurotani fingerprints. For (a) and (b), blank spaces indicate that the correlation was not statistically significant (P-value < 0.05). For (c), blank spaces indicate that the correlation was either not statistically significant or was not strong (|r| ≥ 0.6). We opted to only show strong correlations for (c) due to the large size of the Morgan features (184).

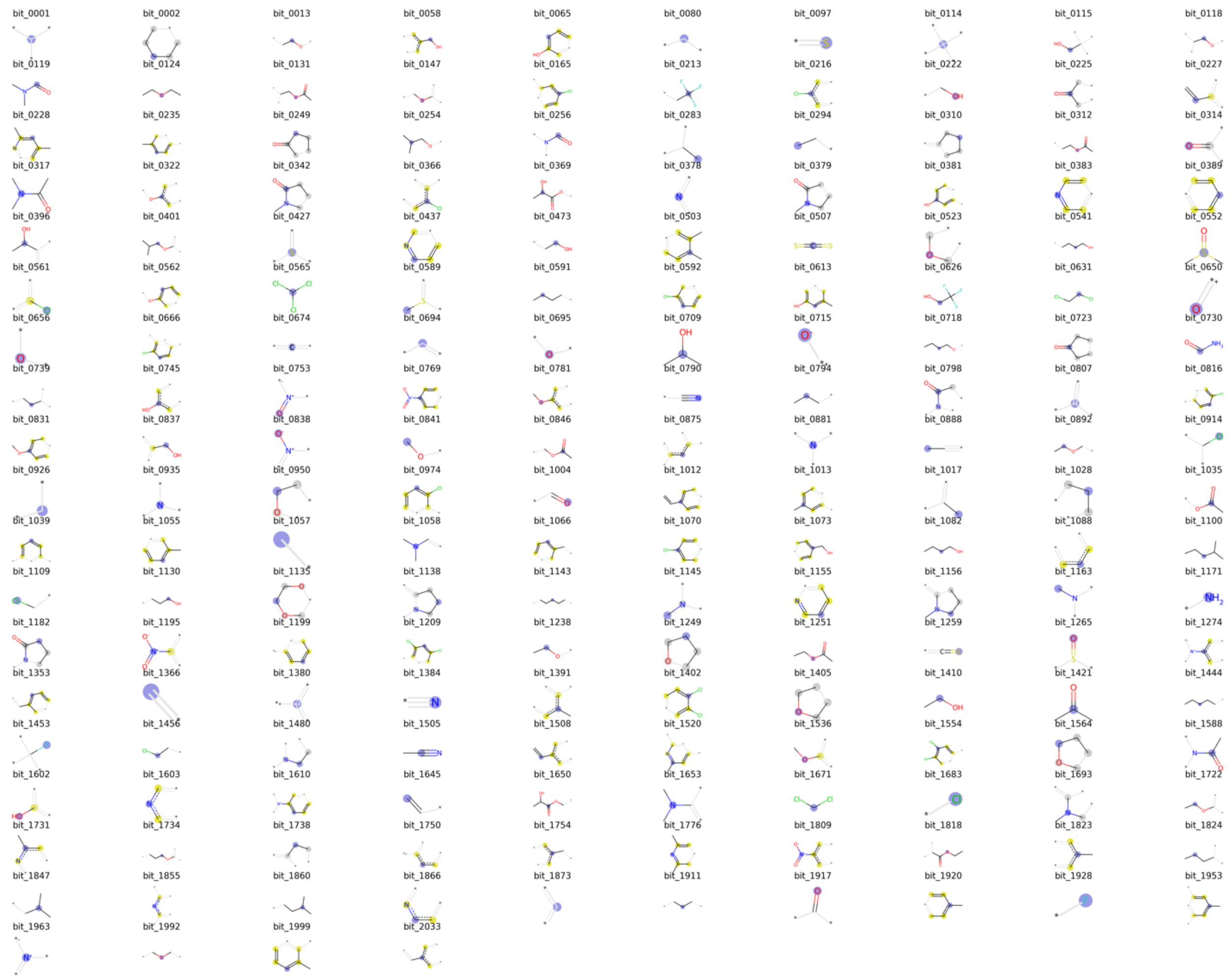

Figure S6. Graphical representation of each Morgan bit. The bit label is placed above each image.

Table S4.Hyperparameters searched during tuning. For the neural network, the activation, density and dropout were searched on a per-layer basis.

Model	Hyperparameters	Values
XGBoost	max_depth	1, 3, 5, 7, 9
	min_child_weight	1, 3, 5
	subsample	0.6, 0.7, 0.8, 0.9, 1.0
	colsample_bytree	0.6, 0.7, 0.8, 0.9, 1.0
	learning_rate	0.01, 0.1, 0.2, 0.3, 0.5
	n_estimators	50, 100, 150, 200, 250
Random Forest	max_depth	1, 3, 5, 7, 9, 50, 100, None
	max_features	0.5, 0.75, “log 2”, “sqrt”
	n_estimators	50, 100, 150, 200, 250
	min_samples_leaf	2, 4, 6
Neural Network	num_layers	2, 3
	layer_activation	"relu”, "tanh", "gelu", "prelu"
	layer_density	50 to 500 in steps of 50
	layer_dropout	0 to 0.7 in steps of 0.05

Table S5. Optimal hyperparameters found for each model.

Model	Hyperparameters	Value
XGBoost	max_depth	7
	min_child_weight	5
	subsample	0.8
	colsample_bytree	0.6
	learning_rate	0.1
	n_estimators	200
Random Forest	max_depth	None
	max_features	0.5
	n_estimators	100
	min_samples_leaf	4
Neural Network	num_layers	2
	layer_activation	“tanh”, “tanh”
	layer_density	500, 350
	layer_dropout	0.3, 0.65

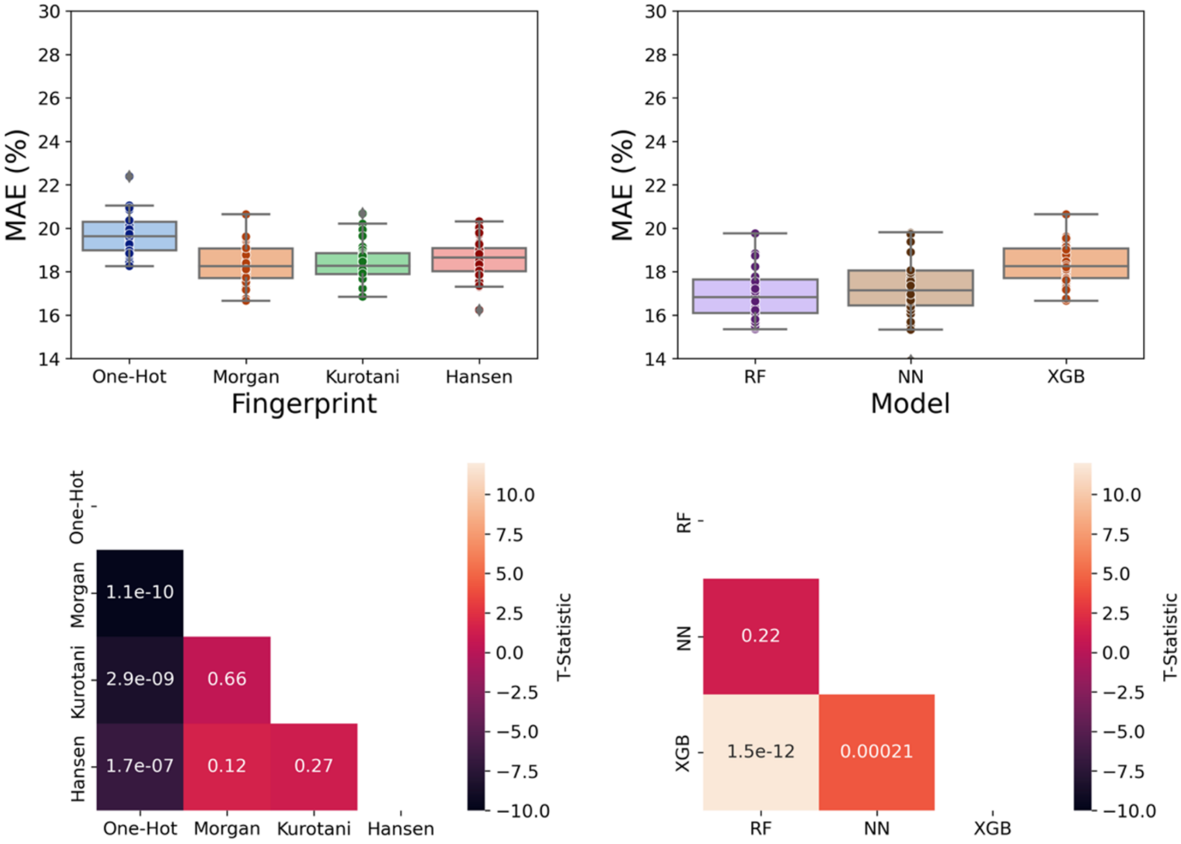

Figure S7. Box and whisker plots comparing the performance of XGB models (a) using different fingerprints and (b) employing Morgan fingerprints with the three different models. Each individual dot represents the test score from one of the 30 50/50 train-test splits. In this scenario, the test set included data points with transmission values predominantly clustered around 0% and 100% in addition to the in-between regions. Additionally, heatmaps display the T-statistic calculated from pair tests of different fingerprints (c) and different models (d). Annotations within the heatmaps correspond to the P-values derived from the pair tests. The label 'Morgan' in (a) and (c) corresponds to the label 'XGB' in (b) and (d).

Additionally, we assessed whether the assumption of normality needed for the t-test is met by taking the difference between MAEs for paired tests for the three models used in figure 3 (a). While the distribution of XGB-NN differences may exhibit slight right-skewness, we argue that this approach remains the most suitable for comparing model architectures.

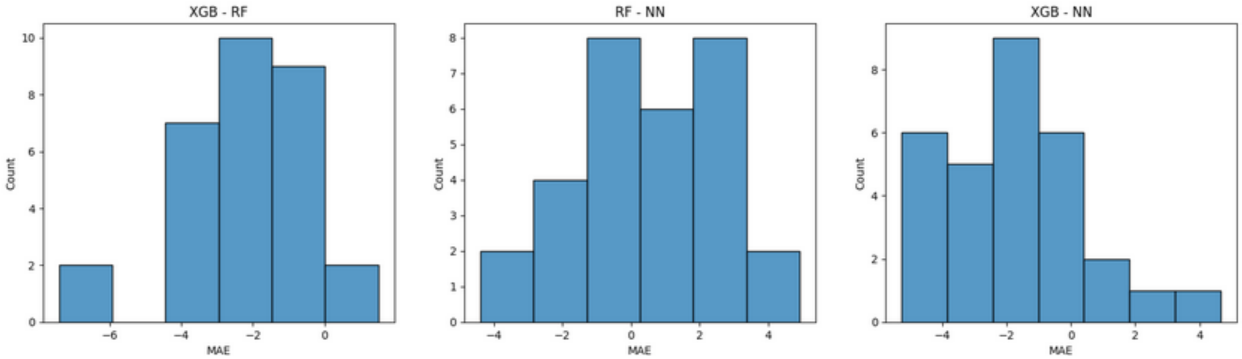

Figure S8: MAE for paired tests of the three models assessed.

E. <u>Feature analysis</u>

To analyze the Morgan fingerprints, we utilized XGBoost's built-in feature importance to ascertain the importance of various features. Figure S9 (a), which plots the mean and standard deviation of the various features across the five-fold models, illustrates that polymer fingerprints consistently emerged as the most crucial features. This prominence is likely attributable to the substantial volume of encoded information within these singular features. Notably, the importance attributed to polymer features appeared to correlate with the quantity of associated data points. Polyethylene terephthalate (PET) and polypropylene (PP) exhibited the highest importance scores, likely owing to their extensive representation in the dataset. Conversely, polylactic acid (PLA) received lower importance ratings, reflecting its limited presence in experimental evaluations. Additionally, PET's high importance may be attributed to its consistent insolubility, allowing the model to reliably associate PET instances with insolubility.

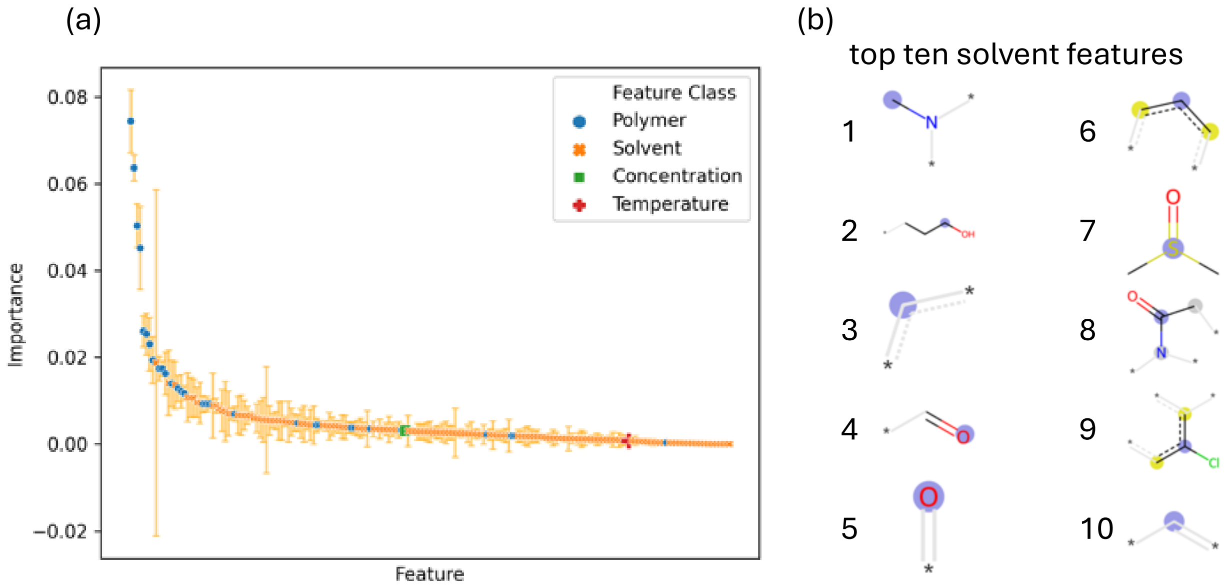

Figure S9. (a) Plot of feature average importance for the five-fold XGB Morgan fingerprint models. Orange bars indicate the standard deviation and colored markers the average for each feature. Blue circles represent polymer one-hot encodings, orange X's solvent Morgan fingerprints, the green square the concentration, and the red cross the temperature. (b) Top ten Morgan fingerprints by average importance.

In our analysis, neither concentration (represented by the green square) nor temperature (indicated by the red cross) emerged as significantly more important than molecular features. This consistency was observed across the various models, as indicated by the remarkably low standard deviation for these features. This is likely because specific combinations of polymers and solvents inherently dictate solubility, irrespective of temperature or concentration variations. However, it's worth noting that concentration did receive a slightly higher weighting than temperature. This emphasis on concentration may stem from its substantial impact: once a certain concentration threshold is reached, polymers tend to either dissolve if the concentration is very low or become insoluble if it's very high, regardless of temperature fluctuations within the range we tested.

The top ten most crucial Morgan fingerprints identified by the models are shown in Figure S9 (b). Among these, features 7 and 8 are exclusively linked to one solvent, while features 1, 2, 4, and 9 are associated with two solvents, and feature 10 with three. This observation suggests that these features may function akin to pseudo one-hot encoders for the solvents in our dataset. This observation can also account for the notably large error bar associated with feature 1. When this feature is absent from a split in the data, it's assumed to have zero importance, thus contributing to the vast error range. Conversely, when feature 1 does come into play, its significance is greatly amplified.

On the other hand, features 3, 5, and 6 exhibit associations with 10 or more solvents, hinting at the possibility that the models are capturing unique insights into the underlying chemistry. Notably, it's intriguing to observe that the selected features predominantly involve aromatic components and carbonyls, both well-known for their significant roles in solubility phenomena. This underscores the models' capacity to discern chemically relevant features critical for understanding solubility behavior.

F. Confusion matrix evaluation of model run with additional partial solubility data

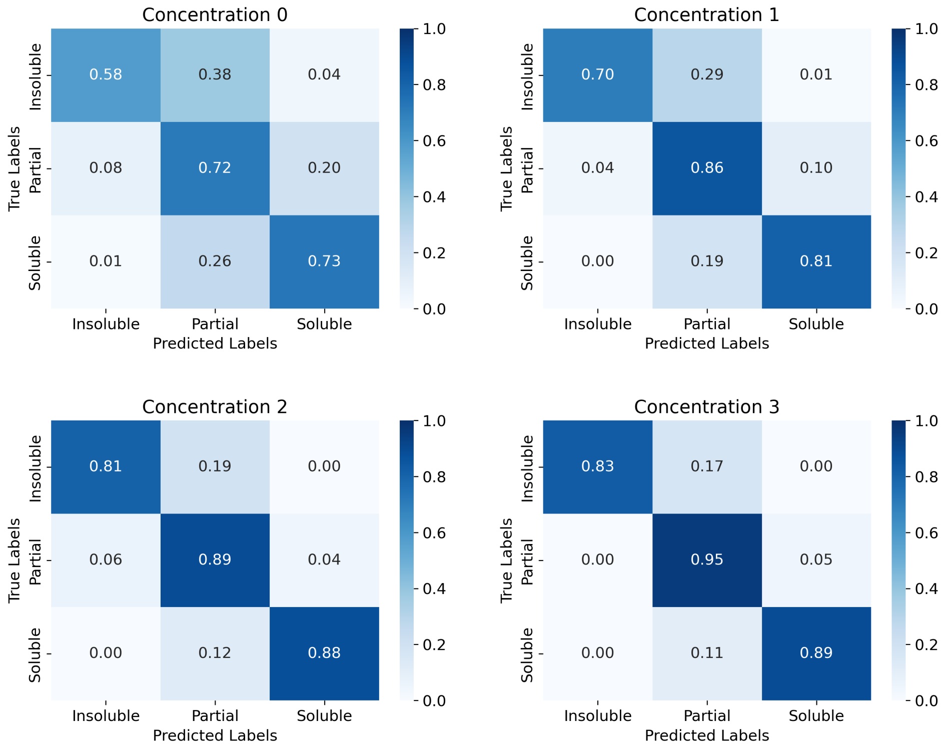

Figure S10. Confusion matrix illustrating the classification performance of the model when 44 unique combinations were added to the database. The regression data has been classified into three categories: 'Insoluble' (transmission < 10%), 'Soluble' (transmission > 85%), and 'Partial' (transmission between 10% and 85% inclusive)."Panels (a) through (d) illustrate model performance as increasing instances of polymer-solvent combinations are observed: (a) - No prior instances, (b) - One instance, (c) - Two instances, and (d) - Three instances.

References:

[1] A. Kurotani, T. Kakiuchi, and J. Kikuchi, "Solubility Prediction from Molecular Properties and Analytical Data Using an In-phase Deep Neural Network (Ip-DNN)," *ACS Omega*, vol. 6, no. 22, pp. 14278–14287, Jun. 2021, doi: 10.1021/acsomega.1c01035.

[2] S. A. Wildman and G. M. Crippen, "Prediction of Physicochemical Parameters by Atomic Contributions," *J. Chem. Inf. Comput. Sci.*, vol. 39, no. 5, pp. 868–873, Sep. 1999, doi: 10.1021/ci990307l.

[3] "RDKit." Accessed: Mar. 08, 2024. [Online]. Available: https://www.rdkit.org/

[4] "HSPiP | Hansen Solubility Parameters." Accessed: Mar. 08, 2024. [Online]. Available: https://www.hansen-solubility.com/HSPiP/
[5] H. L. Morgan, "The Generation of a Unique Machine Description for Chemical Structures-A Technique Developed at Chemical Abstracts Service.," *J. Chem. Doc.*, vol. 5, no. 2, pp. 107–113, May 1965, doi: 10.1021/c160017a018.
[6] D. Rogers and M. Hahn, "Extended-Connectivity Fingerprints," *J. Chem. Inf. Model.*, vol. 50, no. 5, pp. 742–754, May 2010, doi: 10.1021/ci100050t.
[7] D. Mathieu, "Pencil and Paper Estimation of Hansen Solubility Parameters," *ACS Omega*, vol. 3, no. 12, p. 17049, Dec. 2018, doi: 10.1021/acsomega.8b02601.
